# Differential TGFβ pathway targeting by miR-122 in humans and mice affects liver cancer metastasis

**DOI:** 10.1038/ncomms11012

**Published:** 2016-03-18

**Authors:** Shenyi Yin, Yu Fan, Hanshuo Zhang, Zhihua Zhao, Yang Hao, Juan Li, Changhong Sun, Junyu Yang, Zhenjun Yang, Xiao Yang, Jian Lu, Jianzhong Jeff Xi

**Affiliations:** 1 State Key Laboratory of Natural and Biomimetic Drugs, Department of Biomedical Engineering, College of Engineering, Peking University, Beijing 100871, China; 2 State Key Laboratory of Biomembrane and Membrane Biotechnology, Institute of Molecular Medicine, Peking University, Beijing 100871, China; 3 School of Pharmaceutical Sciences, Peking University, Beijing 100191, China; 4 State Key Laboratory of Proteomics, Genetic Laboratory of Development and Diseases, Institute of Biotechnology, Beijing 100071, China; 5 College of Life Science, Peking University, Beijing 100871, China

## Abstract

Downregulation of a predominantly hepatocyte-specific miR-122 is associated with human liver cancer metastasis, whereas miR-122-deficient mice display normal liver function. Here we show a functional conservation of miR-122 in the TGFβ pathway: miR-122 target site is present in the mouse but not human *TGFβR1*, whereas a noncanonical target site is present in the *TGFβ1* 5′UTR in humans and other primates. Experimental switch of the miR-122 target between the receptor *TGFβR1* and the ligand *TGFβ1* changes the metastatic properties of mouse and human liver cancer cells. High expression of TGFβ1 in human primary liver tumours is associated with poor survival. We identify over 50 other miRNAs orthogonally targeting ligand/receptor pairs in humans and mice, suggesting that these are evolutionarily common events. These results reveal an evolutionary mechanism for miRNA-mediated gene regulation underlying species-specific physiological or pathological phenotype and provide a potentially valuable strategy for treating liver-associated diseases.

## Abstract (web summary)

MiR-122 levels correlate with metastasis in human liver cancer but not in mouse models. Here the authors show that miR-122 targets TGFßR1 in mice but TGFß1 in humans, that swapping this specificity affects metastasis, and that many other receptor-ligand pairs are differentially targeted by miRNAs across species.

## Graphical abstract

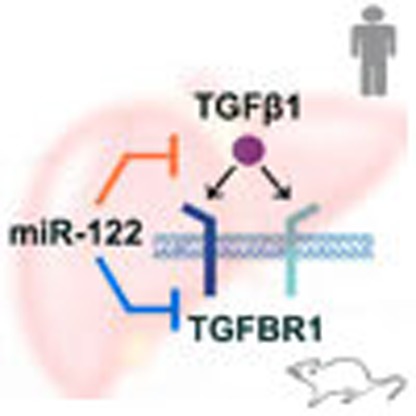

MicroRNAs (miRNAs) are small, endogenous, noncoding RNAs that are 21–24 nucleotides in length and direct the posttranscriptional regulation of gene expression. To date, over 1,800 precursor miRNAs have been identified in the human genome, and they may target 60% of all mammalian genes. This type of small RNAs is well conserved in eukaryotic organisms and is thought to be a vital and evolutionarily ancient component of genetic regulation^1^,^2^. Unlike other signalling modulators, a single miRNA regulates a group of functionally related genes, thus acting more like a function regulating molecule^3^,^4^. These small endogenous RNAs have been implicated in a wide range of important biological processes, such as cell growth and differentiation as well as development^2^. Aberrant expression of miRNAs has been implicated in numerous disease states, including global dysregulation that occurs in human cancer^5^,^6^,^7^,^8^,^9^,^10^,^11^.

Primary liver cancer is the fifth most frequently diagnosed cancer globally and the second leading cause of cancer death^12^,^13^. MiR-122 is a liver-specific miRNA that constitutes 70% of the liver miRNA population. A number of studies have reported that miR-122 plays a critical role in maintaining the adult liver phenotype or in regulating cholesterol biosynthesis. The downregulation of miR-122 is associated with human hepatocellular carcinoma (HCC) metastasis, poor prognosis and reduced survival time^14^,^15^. In addition, it was found that miR-122 is essential to the stability and propagation of hepatitis C virus (HCV) RNA^16^. A phase II study of targeting miRNA-122 to treat HCV infection was reported recently^17^. These results demonstrated that miR-122 is an invaluable target against liver-associated diseases.

However, unlike in the human case, HCC was not observed in the miR-122 knockdown mouse experiments^18^,^19^. Even in the mice with knockout of the *Mir-122* locus, <7% (3/39) developed metastatic lung nodules^20^. At this time, there is no direct evidence for an miRNA mechanism responsible for these paradoxical results in liver cancer development in humans and mice that have been reported. The tumour growth factor β (TGFβ) signalling pathway is conserved from flies to humans and is involved in many cellular processes, such as cell growth, cell differentiation, apoptosis, embryo development, cellular homeostasis and other cellular functions. The regulation pathway is relatively simple. Once the binding of TGFβ to either type II or type III receptors results in the activation of type I receptors via phosphorylation, then further phosphorylating Smad2 or Smad3, which then binds to Smad4. The resulting Smad complex then moves into the nucleus, in which it interacts with various transcription factors in a cell-specific manner to regulate the transcription of many genes. A large body of literature has underscored that the TGFβ signalling pathway plays a critical role in liver metastasis^21^,^22^. Here we report a new species-dependent miRNA signalling mechanism and propose that switch of miR-122 target from *TGFβR1* to *TGFβ1* underlies the different patterns of liver cancer metastasis between the two species.

## Results

### miR-122 inhibits TGFβ1 in humans but TGFβR1 in mice

**Figure 1:**
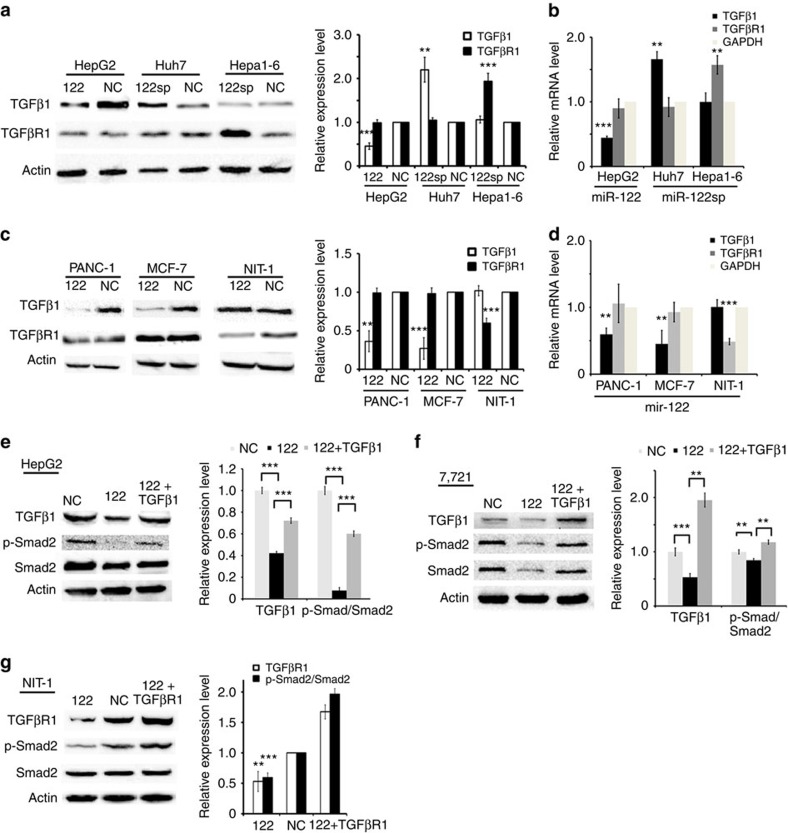
miR-122 inhibits TGFβ1 in human cells, but TGFβR1 in mouse cells. (**a**) Western blot analysis of TGFβ1 and TGFβR1 in HepG2, Huh7 or Hepa1-6 cells when treated with an miR-122 expression plasmid (122), miR-122 sponge (122sp) or scramble sequence as an negative control (NC), respectively. Quantitative analysis is shown on the right, and three independent repeats are performed in each experiment. HepG2 and Huh7 are human liver cancer cells, while Hepa1-6 is mouse cell line. (**b**) Real-time PCR analysis of *TGFβ1* and *TGFβR1* mRNAs in either HepG2, Huh7 or Hepa1-6 cells treated with miR-122, miR-122sp or NC. Three independent repeats are performed in each experiment. (**c**) Western blot analysis of TGFβ1 and TGFβR1 in PANC-1 or MCF-7 cells as well as NIT-1 cells transfected with a miR-122 expression plasmid or NC, respectively. Quantitative analysis is shown on the right. *n*=3. PANC-1 or MCF-7 are human non-liver cell line, while NIT-1 is a mouse pancreatic β-cell line. (**d**) Real-time PCR analysis of *TGFβ1* and *TGFβR1* mRNAs in PANC-1 or MCF-7 cells as well as NIT-1 cells treated with miR-122 or NC. Three independent repeats are performed in each experiment. (**e**) Western blot analysis of intracellular TGFβ1, Smad2 and p-Smad2 in HepG2 cells transfected with miR-122, NC or miR-122 together with TGFβ1, respectively. Quantitative analysis is shown on the right. Three independent repeats are performed in each experiment. (**f**) Western blot analysis of intracellular TGFβ1, Smad2 and p-Smad2 in SMC-7721 cells transfected with miR-122, NC or miR-122 together with TGFβ1. Quantitative analysis is shown on the right, and three independent repeats are performed in each experiment. (**g**) Western blot analysis of intracellular TGFβR1, Smad2 and p-Smad2 in NIT-1 cells transfected with miR-122, NC or miR-122 together with TGFβR1. Quantitative analysis is shown on the right, and three independent repeats are performed in each experiment. Error bars, ±s.d. **P<0.01; ***P<0.001 by two-sided Student's *t*-test. β-Actin was used as a loading control.

We first assessed the effect of miR-122 on the endogenous levels of TGFβ1 in three liver cell lines. HepG2 and Huh7 are human liver cancer cell lines, whereas Hepa1-6 cells are mouse liver cancer cells. HepG2 has a low level of miR-122 expression, whereas both Huh7 and Hepa1-6 have a high level (Supplementary Fig. 1a). HepG2 cells were transfected with the miR-122 overexpression plasmid (transient expression). The ago protein immunoprecipitation (AGO-IP) assay showed that the miR-122 expression level increased 60-fold, while the expression of TGFβ1 decreased to 54% (Fig. 1a; Supplementary Fig. 1b)^23^. Consistent with this, when Huh7 cells were treated with the miR-122 sponge expression construct, there was a twofold increase in TGFβ1 expression (Fig. 1a; Supplementary Fig. 1c). However, the silencing of miR-122 in Hepa1-6 cells resulted in no change of TGFβ1, but a twofold increase in TGFβ receptor 1 (TGFβR1) expression (Fig. 1a; Supplementary Fig. 1d). In contrast, the expression of TGFβR1 remained unchanged in response to miR-122 or its sponge in human liver cancer cells (Fig. 1a). We also investigated the change of TGFβ1 or TGFβR1 messenger RNAs (mRNAs) when miR-122 was increased or decreased in human or mouse cell lines, and found the similar pattern to their protein one (Fig. 1b).

To further investigate whether miR-122 targets *TGFβ1* in humans, but *TGFβR1* in mice, we analysed four more liver cell lines When two human liver cell lines, SMMC-7721 and LM9, were transfected with the miR-122 overexpression plasmid, both transcriptional and translational expression levels of TGFβ1 were decrease by over 50% and the levels of TGFβR1 remains unchanged. Consistent with the data in Hepa1-6 cells, the overexpression of miR-122 in two mouse liver cell lines, H22 and NCTC1469, resulted in the decrease of *TGFβR1* in both protein and mRNA levels, but no change of *TGFβ1* (Supplementary Fig. 1e,f).

We then investigated whether the orthogonal effect of miR-122 is limited to liver cells. Two human cell lines were examined, including PANC-1 (human pancreatic carcinoma, an epithelial-like cell line) and MCF-7 (a human breast adenocarcinoma cell line). The overexpression of miR-122 in these two cell types resulted in a decrease of the TGFβ1 level by 80% and 70%, respectively, and no change in the expression level of TGFβR1 (Fig. 1c). When miR-122 was overexpressed in NIT-1 cells, which is a pancreatic β-cell line established from a transgenic non-obesity diabetes (NOD/Lt) mouse, the expression level of TGFβR1 was decreased by 40% and the expression level of TGFβ1 was unchanged. The change of TGFβ1 or TGFβR1 mRNAs demonstrated the similar pattern to their protein one in these non-liver cell lines (Fig. 1d), indicating the mechanism of mRNA degradation by miR-122.

We next studied whether the repressive effect of miR-122 is specific to TGFβ isoforms or passes on the downstream signalling components. miR-122 was overexpressed in HepG2 cells to generate a stable cell line, which is referred to as HepG2-122. The quantitative examination showed that the TGFβ1 protein level was decreased in HepG2-122 cells, whereas the expression levels of TGFβ2 or TGFβ3 did not exhibit any significant changes in HepG2-122 cells compared with HepG2 cells (Supplementary Fig. 1g). Western blot analysis showed that the ratio of the p-Smad2 to Smad2 level was decreased by up to 50% in HepG2-122 cells. When TGFβ1 was overexpressed in HepG2-122 cells, the ratio recovered close to the control level (Fig. 1e). Furthermore, the similar results were found in another human liver cancer cells, SMMC-7721, when transfected with miR-122 or miR-122 together with TGFβ1 (Fig. 1f). Similarly, the ratio of the p-Smad2 to Smad2 level was inhibited when miR-122 was overexpressed in NIT-1 cells, and the decreased ratio was reversed by TGFβR1 (Fig. 1g). Together, these data demonstrate that miR-122 inhibits *TGFβ1* in humans, but *TGFβR1* in mice.

### miR-122 targets TGFβ1 5′UTR in humans

**Figure 2:**
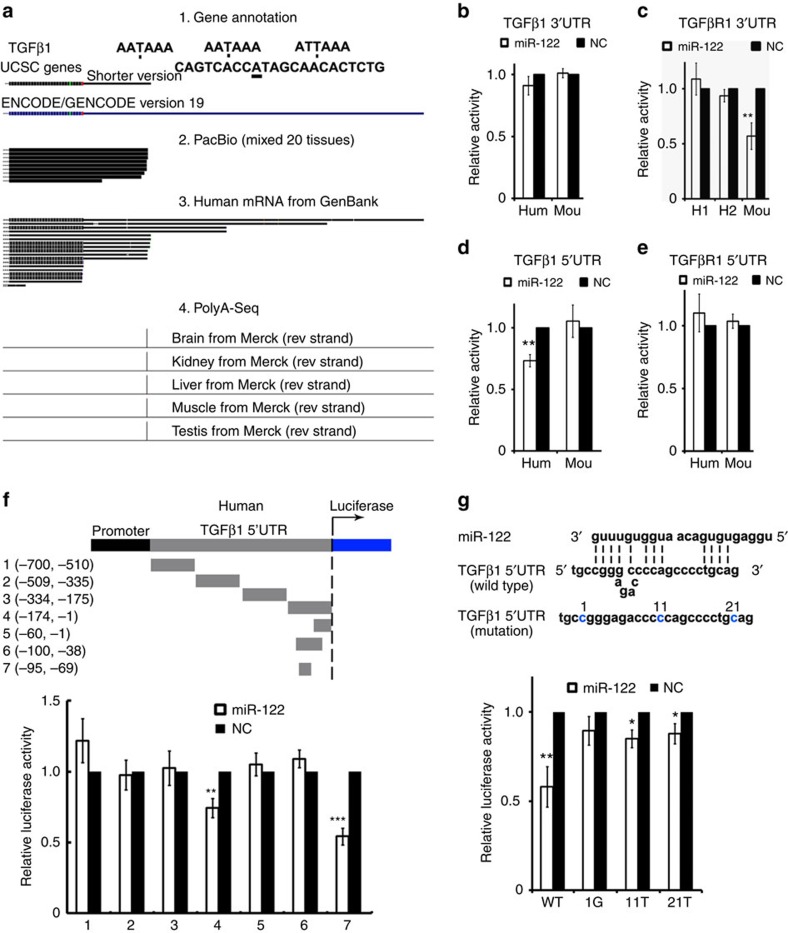
miR-122 targets human *TGFβ1* 5′UTR in a non-‘seed-region' base-pairing manner. (**a**) Evidence for the different versions of human *TGFβ1* mRNA, which include gene annotation from UCSC Genes and ENCODE/GENCODE, the PacBio sequencing data from mixed 20 tissues^24^, human mRNA from GenBank (NCBI) or PolyA sequencing data of different tissues^25^. (**b**–**e**) Luciferase activity was measured after transfection of the indicated reporter constructs in Hela cells. *TGFβR1* 5′UTR was cloned into the promoter region. Hum, human, Mou, mouse. The *TGFβ1* 3′UTRs were cloned into the 3′UTR of luciferase in a pGL plasmid. The 3′UTR of human *TGFβ1* was divided into two parts, H1 or H2, because of its long length. Six independent repeats are performed in each experiment. (**f**) Luciferase activity was measured after transfection of the indicated reporter constructs. The human *TGFβ1* 5′UTR was divided into three different fragments and then each truncated 5′UTR was cloned into the promoter region of pGL plasmid. Six independent repeats are performed in each experiment. (**g**) Diagram depicting the miR-122 targeting region of the *TGFβ1* 5′UTR and the mutated site. Luciferase reporter activity containing the different constructs was shown below. Error bars, ±s.d. *P<0.05; **P<0.01; ***P<0.001 by two-sided Student's *t*-test.

We first made comparison of *TGFβ1* or *TGFβR1* 3′-untranslated region (UTR) conservation sequences in humans and mice (Supplementary Fig. 2a), and constructed a group of luciferase reporters (Supplementary Fig. 2b). A miR-122 target site might exist downstream of human *TGFβ1* on the basis of the prediction of microcosm, but substantial evidence showed that this site is outside the 3′UTR in most cases^24^,^25^ (Fig. 2a). In addition, the data regarding the 3′UTR in the rhesus monkey demonstrated that only the short 3′UTR exists, which excludes the predicted target site (Supplementary Fig. 2c). Thus, a short 3′UTR was cloned into a luciferase reporter and characterized (Fig. 2a). The reporter containing the 3′UTR of mouse *TGFβR1* was decreased to 60% by miR-122 treatment, whereas the reporter containing the 3′UTR of human *TGFβ1* or *TGFβR1* was unchanged, indicating that there is no target site in their 3′UTRs (Fig. 2b,c).

A few of studies showed that a miRNA target site may exist in the 5′UTR of a certain gene^26^,^27^, we next cloned the full length of human or mouse 5′UTR into a luciferase reporter (Supplementary Fig. 2d). Only the reporter containing the 5′UTR of human *TGFβ1* was decreased to 75% by miR-122 (Fig. 2d,e). To identify the exact targeting site, we then constructed a series of luciferase reporters containing differently truncated versions of the human 5′UTR. We found that the reporters containing the fourth fragment or seventh fragment were silenced by miR-122 (Fig. 2f). Unexpectedly, the reporter containing the sixth fragment was resistant to the silence of miR-122. We hypothesized that a RNA secondary structure may exist in the sixth fragment due to the high GC content (Supplementary Fig. 2e), which was supported by a swap-mutation experiment (Supplementary Fig. 2f). Subsequent mutation experiments, in which three nucleotides most affecting the stability of the predicted RNA secondary structure was mutated one by one, identified the exact targeting sequence, which may pair to the 3′ region of miR-122, rather than to its seed sequence (Fig. 2g). Thus, miR-122 directly targets a noncanonical site in *TGFβ1* 5′UTR in humans, but it targets *TGFβR1* in mice.

### A conserved mechanism of miR-122 switching targets

**Figure 3:**
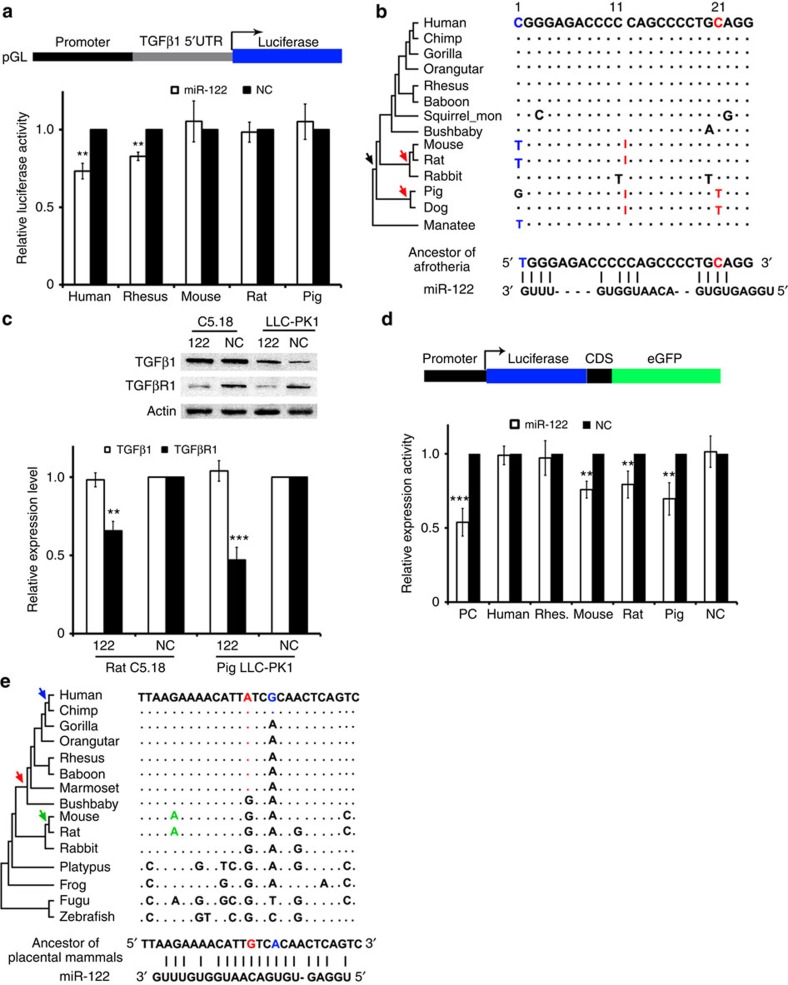
Evolutionary analysis of miR-122 targeting *TGFβ1/TGFβR1* in vertebrates. (**a**) Luciferase activity was measured after transfection of the indicated reporter constructs. *TGFβ1* 5′UTR was cloned into the promoter region of a pGL plasmid. Rhesus, Rhesus monkey. Six independent repeats are performed in each experiment. (**b**) The evolutionary trajectory of the miR-122 target site in the *TGFβ1* 5′UTR in animals. The gain of the miR-122 target site occurs in the common ancestor of the manatee and humans as well as other primates (black arrow), while the loss of this site in the pig, dog, rat or mouse due to the insertion of a few of bases between the 11th and 12th bases (red arrow). The dot means the nucleotide is identical to the one in humans, and the red line means the insertion of one or a few of bases. For the predicted miR-122 target site in each species, the luciferase assay was performed. ‘+' denotes the silence effect, and ‘−' denotes no silence effect. Experimental data were shown in Supplementary Fig. 3d. (**c**) Expression levels of TGFβ1 or TGFβR1 in C5.18 (rat cells) or LLC-PK1 (pig cells) transfected with an miR-122 expression plasmid or NC. Quantitative analysis is shown below and three independent repeats are performed in each experiment. (**d**) Luciferase activity was measured after transfection with the indicated reporter constructs. The candidate sequences (see Supplementary Table 2 for the sequences) were cloned in the CDS of a fusion protein of luciferase and eGFP. NC, negative control; PC, positive control; Rhes., Rhesus monkey. Six independent repeats are performed in each experiment. (**e**) The evolutionary trajectory of the miR-122 target site in the *TGFβR1* CDSs in animals. Three events are involved in the gain and loss of target sites, that is, a G–>A mutation (red), A–>G mutation (blue) and a G–>A mutation (green). The dot means the nucleotide is identical to the one in humans. For the predicted miR-122 target site in each species, the luciferase assay was performed. ‘+' denotes the silence effect, and ‘−' denotes no silence effect. Experimental data were shown in Supplementary Fig. 3f. Error bars, ±s.d. **P<0.01; ***P<0.001 by two-sided Student's *t*-test.

Since the sequence of miR-122 is identical in vertebrates, we performed an analysis of the degree of conservation of miR-122 target sites in *TGFβ1/TGFβR1* in different species. First, we experimentally determined whether miR-122 targets *TGFβ1/TGFβR1* in the rhesus monkey, pig or rat. We cloned the 5′UTR and 3′UTR, and constructed them into a luciferase reporter, respectively (Fig. 3a; Supplementary Fig. 3a–c). miR-122 significantly silenced the reporter containing the rhesus monkey *TGFβ1* 5′UTR, which contained a target sequence exactly the same as that found in humans (Fig. 3b; Supplementary Table 1). We then performed a comparative genomic analysis of the sequence of this target site across representative species in the animal phylogenetic tree. miR-122 significantly inhibited the reporter containing the *TGFβ1* 5′UTR of the manetee, in which 1C is changed into 1T (Fig. 2g). The conversion of 21C to 21T, accompanied by the insertion of either one or a small number of bases between the 11th and 12th bases, resulted in a total loss of the inhibition by miR-122, such as those found in the mouse, rat, dog and pig (Fig. 3b; Supplementary Fig. 3b,d). No homologous sequence was identified in the *TGFβ1* 5′UTR of the more distantly related vertebrates, such as the birds or fish, indicating that the gain of the miR-122 target site occurs in the common ancestor of the *Afrotheria* and *Primate*.

Surprisingly, no miR-122 target site was identified in either the *TGFβ1* or *TGFβR1* UTRs in pigs or rats (Fig. 3a; Supplementary Fig. 3a–c). However, western blot assay demonstrated that miR-122 inhibits the expression level of *TGFβR1* in rat or pig cell lines(Fig. 3c). We then switched our attention to coding sequences (CDS), in which a group of candidate miR-122 target sites were identified (Supplementary Table 2). The sequences were cloned into the middle of a reporter construct expressing a luciferase–green fluorescent protein (GFP) fusion protein, respectively (Fig. 3d). Luciferase assays proved that a miR-122 target site exists in the *TGFβR1* CDSs of pigs or rats, but not humans or monkeys. These findings were further supported by fluorescence assays (Supplementary Fig. 3e).

The target sites in the *TGFβR1* CDSs are highly conserved in all vertebrates (Supplementary Table 2). By comparative genomic analysis of the miR-122 target sites across representative species in the animal phylogenetic tree, we traced the evolutionary trajectory. On the basis of parsimonious inference, we found that three events are involved in the gain and loss of target sites (Fig. 3e). First, during the split between the bushbaby and the most recent common ancestor of humans and the marmoset, a G–>A mutation (red in Fig. 3e) occurred in the most recent common human and marmoset ancestor, generating the allele that is presently preserved in monkeys and other primate species. This mutation abolishes a pairing between miR-122 and the target site. Furthermore, in the most recent common ancestor of humans and the chimpanzee, a second mutation (A–>G, blue in Fig. 3e) occurred, and this mutation further destroyed the binding affinity of miR-122 and the target sites. On the other hand, in the most recent ancestor of mouse and rat, a G–> A mutation (green in Fig. 3e) created a pairing site for miR-122, which in principle enhances the binding specificity of miR-122 and the target site. These analyses were further supported by fluorescence assays (Supplementary Fig. 3f). Therefore, this is a good demonstration of the evolutionary scenario in which the *TGFβR1* CDS regulated by miR-122 in the mouse evolved to the *TGFβ1* 5′UTR in humans.

### Distinct metastatic traits by miR-122 loss in humans or mice

miR-122 levels are reduced in clinical samples of HCC^14^,^15^. Given that miR-122 is a liver-specific molecule, the TGFβR1 increase was constrained to liver cancer cells. However, as a secreted protein, TGFβ1 regulates the functions of neighbouring cells. We thus hypothesized that the loss of miR-122 in liver cancers would generate distinct pathological effects in humans and mice, mainly with regard to tumour metastasis-relevant traits.

**Figure 4:**
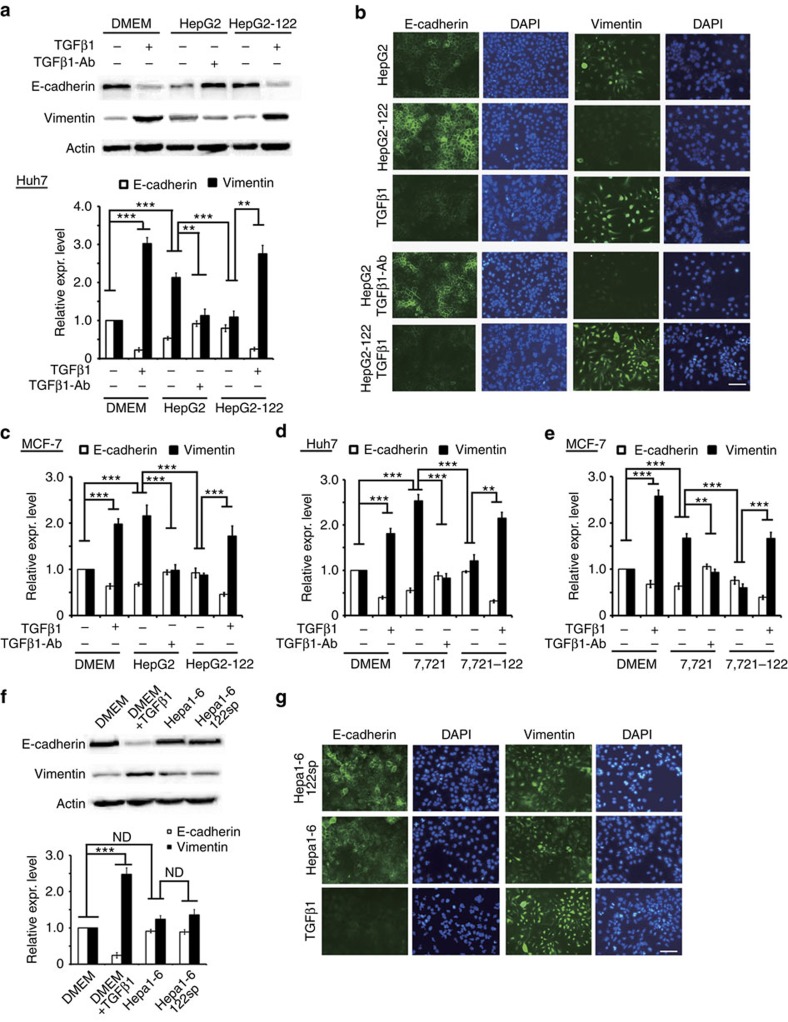
Differential targeting of *TGFβ1/TGFβR1* is the underlying reason for the distinct impact of miR-122 on EMT in human or mouse cells. (**a**) Western blot analysis of E-cadherin and vimentin levels in Huh7 treated as indicated. HepG2 and HepG2-122 stands for the supernatant of HepG2 and HepG2-122 cells, respectively. Quantitative analysis is shown on the right. Three independent repeats are performed in each experiment. (**b**) E-cadherin and vimentin were used as markers of EMT in Huh7 cells treated as indicated. 4,6-Diamidino-2-phenylindole (DAPI) staining was used to detect nuclei. Scale bar, 50 μm. (**c**) Quantitative analysis of E-cadherin and vimentin levels in MCF-7 treated as indicated. HepG2 and HepG2-122 stands for the supernatant of HepG2 and HepG2-122 cells by western blot, respectively. Three independent repeats are performed in each experiment. (**d**) Quantitative analysis of E-cadherin and vimentin levels in Huh7 treated as indicated. 7721 and 7721-122 stands for the supernatant of SMC-7721 and SMC-7721-122 cells by western blot, respectively. Three independent repeats are performed in each experiment. (**e**) Quantitative analysis of E-cadherin and vimentin levels in MCF-7 treated as indicated. 7721 and 7721-122 stands for the supernatant of SMC-7721 and SMC-7721-122 cells by western blot, respectively. Three independent repeats are performed in each experiment. (**f**) Western blot analysis of E-cadherin and vimentin levels in Huh7 treated as indicated. Hepa1-6 and Hepa1-6-122sp stands for the supernatant of Hepa1-6 and Hepa1-6-122sp cells, respectively. Three independent repeats are performed in each experiment. (**g**) E-cadherin and vimentin were used as markers of EMT in Huh7 cells treated as indicated. DAPI staining was used to detect nuclei. Scale bar, 50 μm. Error bars, ±s.d. **P<0.01; ***P<0.001 by two-sided Student's *t*-test.

We first investigated whether a miR-122-mediated repression of *TGFβ1/TGFβR1* affected an epithelial–mesenchymal transition (EMT), one of the initiating steps of primary tumour invasion. The culture supernatants of HepG2 or HepG2-122 cells grown for 48 h were collected. Huh7 cells were treated with these supernatants or recombinant TGFβ1 protein at a final concentration of 2.5 ng ml^−1^. After 24 h, western blot assay showed that the HepG2 culture supernatant, similar to the recombinant TGFβ1 protein, caused a decrease of E-cadherin by 50%, but a twofold increase of vimentin (Fig. 4a). The changes in E-cadherin and vimentin were reversed by the HepG2-122 culture supernatant. To further confirm the effect of miR-122 on EMT was mediated through TGFβ1, HepG2 and HepG2-122 culture supernatants were treated with a TGFβ1 neutralizing antibody (TGFβ1-Ab) and TGFβ1, respectively. It was shown that TGFβ1-Ab overturned the effect of HepG2 supernatant, and TGFβ1 reversed the effect of the HepG2-122 supernatant. These results are consistent with the results of immunofluorescence analysis (Fig. 4b). To further confirm that a miR-122-mediated repression affected EMT in human cells, we demonstrated three more experiments. That is, HepG2 or SMMC-7721 cells were treated with TGFβ1 antibody, miR-122 overexpression or both, and then their supernatants were treated to Huh7 or MCF cells, respectively. Similarly, the overexpression of miR-122 in HepG2 or SMMC-7721 resulted in the increase of E-cadherin as well as the decrease of vimentin, regardless of treating Huh7 or MCF cells (Fig. 4c–e).

Similar experiments were performed with either Hepa1-6 cell culture supernatant or Hepa1-6-122 sponge cell culture supernatant. Neither E-cadherin nor vimentin exhibited any difference in two cases (Fig. 4f,g). In confirmation of the finding that mouse TGFβ1 induced EMT in human cells, when recombinant mouse TGFβ1 was added to the DMEM medium in Huh7 cells, the expression of E-cadherin exhibited a 4-fold decrease and vimentin a 2.5-fold increase.

Vascular endothelial growth factor (VEGF) is an another critical factor in tumour metastasis that is upregulated in response to TGFβ1 stimulation^28^,^29^. We therefore investigated whether miR-122-mediated TGFβ1/TGFβR1 changes have a distinct effect on angiogenesis in humans and mice. The culture supernatant of HepG2 or HepG2-122 cells was used to treat human umbilical vein endothelial cells (HUVECs). The culture supernatant of HepG2 cells caused a 3.5-fold increase in tubular length and 2.0-fold increase of the branch point number (BPN) compared with treatment with DMEM media (Supplementary Fig. 4a). The HepG2-122 culture supernatant reversed the effect of the HepG2 culture supernatant on both tubular length and BPN. Consistent with this finding, TGFβ1-AB blocked the effect of the HepG2 supernatant and TGFβ1 reversed the effect of the HepG2-122 supernatant. Neither Hepa1-6 nor Hepa1-6-122 sponge cell supernatant affected tubular length or BPN (Supplementary Fig. 4b). When the expression of VEGF was examined under the conditions described above using an enzyme-linked immunosorbent assay (ELISA) assay, a decrease in the VEGF level was found in HepG2-122 cells, and this decrease was ameliorated when TGFβ1 was overexpressed (Supplementary Fig. 4c). However, neither Hepa1-6 nor Hepa1-6-122 sponge cells induced any changes in the VEGF level. The luciferase assay further excluded VEGF as a target of miR-122 (Supplementary Fig. 4d). These *in vitro* data showed that miR-122-mediated TGFβ1/TGFβR1 activity generated distinct metastasis-relevant traits in human or mouse cells.

### Species-specific effect of miR-122 on liver cancer metastasis

**Figure 5:**
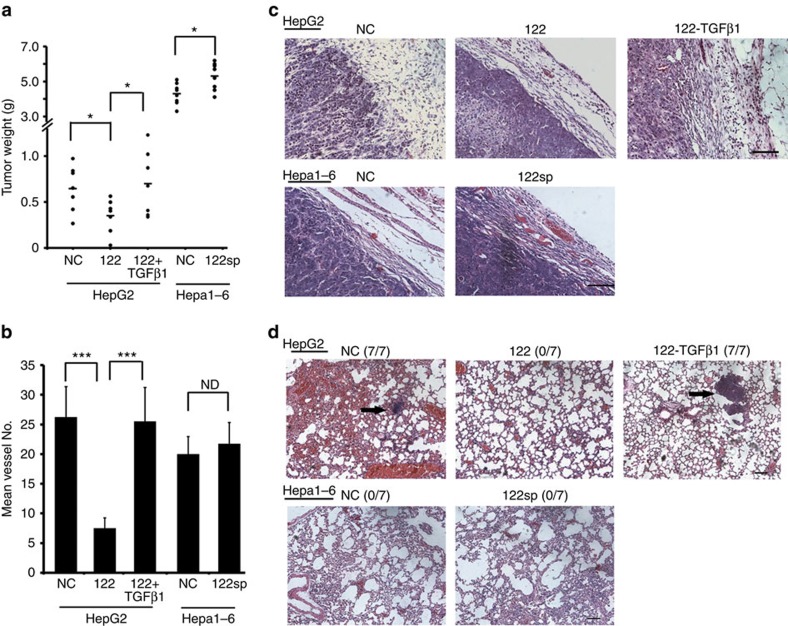
Switch of miR-122 targeting from *TGFβR1* to *TGFβ1* generates different metastatic effects in human xenografts or mouse allografts. (**a**) Tumour volume was measured after 5 weeks when the indicated cells expressing individual miRNAs or the control hairpin (negative control, NC) cells were subcutaneously implanted into immunodeficient mice. Seven independent repeats are performed in each experiment. (**b**) Quantitative analysis of the vessel numbers in the indicated tissues. Seven independent repeats are performed in each experiment. (**c**) Haematoxylin and eosin (H&E) stain of primary tumours 60 days after subcutaneous injection. Mice were subcutaneously implanted with HepG2 cells stably expressing NC, miR-122 or both miR-122 and TGFβ1 (122-TGFβ1), or with Hepa1-6 cells stably expressing NC or miR-122 sponge (122sp) in each group. Seven independent repeats are performed in each experiment. Scale bar, 50 μm. (**d**) H&E stain of lungs isolated from mice that received tail vein injection of the indicated cells. Seven independent repeats are performed in each experiment. Scale bar, 50 μm. Error bars, ±s.d. *P<0.05; ***P<0.001 by two-sided Student's *t*-test. ND, no difference.

We first examined the effect of decreased miR-122 on the development of liver cancer using human xenografts or mouse allografts. The growth weight and metastatic status of the liver tumour samples were compared in the mouse tumour models implanted with different human or mice liver cell lines. The three stably transfected human cell lines HepG2-NC, HepG2-122 and HepG2-122-TGFβ1 were subcutaneously implanted into nude mice. After 5 weeks, mice were killed and the tumours were measured. A significant decrease in tumour weight and size was observed in the HepG2-122 group (Fig. 5a; Supplementary Fig. 5a). We then measured the number of neovessels in these tumours by CD31 staining and detected a fourfold decrease in the generation of new blood vessels in the HepG2-122 tumour (Fig. 5b; Supplementary Fig. 5b). These decreases in both tumour weight and angiogenesis were compromised when *TGFβ1* was overexpressed in HepG2-122 (Fig. 5a,b). We also characterized the effect of miR-122 on the local invasion or distant metastasis in either xenografts or allografts. We found that miR-122 expressing tumours were well encapsulated and non-invasive (Fig. 5c). Examination of the lungs revealed metastatic nodules had developed in the control mice (7/7), but not in the mice implanted with HepG2-122 cells (0/7; Fig. 5d). The overexpression of *TGFβ1* in HepG2-122 reversed the effect of miR-122 on both local invasion and distant metastasis.

Two types of cells, Hepa1-6-NC and Hepa1-6-122 sponge, were subcutaneously implanted into nude mice. We characterized the tumour weight and angiogenic status, as well as local or distant invasion in these two types of mice. Although the tumour weight slightly increased in Hepa1-6-122 sponge group, no significant change between the two groups was observed for any other features (Fig. 5a–d; Supplementary Fig. 5c).

**Figure 6:**
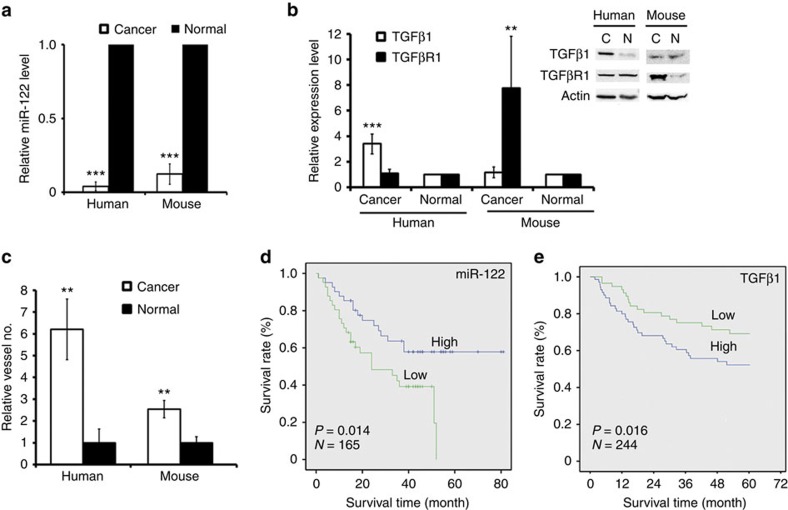
Loss of miR-122 resulted in the different metastatic effects in humans or mice liver cancers *in vivo*. (**a**) Quantitative analysis of miR-122 levels in hepatocellular tumours in HBx gene knock-in transgenic mice or normal liver tissue (eight independent repeats) as well as human hepatocellular tumour tissues or normal adjacent tissue (six independent repeats). (**b**) Western blot assay showing the expression levels of TGFβ1/TGFβR1 in tissues, as indicated. Representative images are shown in the inset. Eleven independent repeats are performed in each experiment. (**c**) Quantitative analysis of vessel numbers in the indicated tissues by CD31 staining. (**d**) Kaplan–Meier curves for overall survival in HCC cohorts from the Liver Cancer Institute (LCI) and Zhongshan Hospital. Expression value=log2 of Robust multi-array analysis (RMA)-calculated signal intensity. miR-122 expression value>0.39 was designed as the high expression group, while miR-122 expression value<-0.58 was designed as the low expression group. *n*=165 patients; *P* value based on the Mantel–Cox log-rank test. (**e**) Kaplan–Meier curves for overall survival in HCC cohorts from the LCI and Zhongshan Hospital. Expression value=log2 of RMA-calculated signal intensity. TGFβ1 expression value <4 was designed as the low expression group, while TGFβ1 expression value>5 was designed as the high expression group. *n*=244 patients; *P* value based on the Mantel–Cox log-rank test. Error bars, ±s.d. **P<0.01; ***P<0.001 by two-sided Student's *t*-test. ND, no difference.

Second, we examined the respective expression levels of miRNA-122, TGFβ1 and TGFβR1 in human and mouse hepatocellular carcinoma samples. Quantitative analysis showed that miR-122 levels were decreased >10-fold in human tumour samples relative to normal adjacent samples (Fig. 6a). Western blot assay showed that the level of TGFβ1 was upregulated approximately four times in 11 liver cancer samples, whereas TGFβR1 expression remained moderate decrease (Fig. 6b; Supplementary Fig. 6a). We investigated the angiogenic status of the liver tissue samples and found a sixfold increase in angiogenesis in the cancer samples compared with normal tissues (Fig. 6c). It was reported that miR-122 repression coincides with the acquisition of a liver invasive phenotype^14^,^15^. To determine whether the expression of miR-122, TGFβ1 or TGFβR1 is associated with human liver cancer metastasis, we examined primary liver tumour gene expression data sets with corresponding disease outcome annotation^30^,^31^,^32^. Within these cohorts, a low expression of miR-122 was associated with poor survival (Fig. 6d). A high expression of TGFβ1 was associated with poor survival compared with lower TGFβ1 expressing tumours; 5-year survival among patients with a lower TGFβ1 expression was 70%, whereas >50% of the patients with higher TGFβ1 expression succumbed to their disease in this period (Fig. 6e). Consistent with the results of our cell studies, the expression of TGFβR1 was not associated with overall survival (Supplementary Fig. 6b, representative data of miR-122, TGF-β1 and TGF-βR1 expression shown in Supplementary Fig. 6c–e).

Eight mice with hepatocellular carcinoma were generated through the knock-in expression of HBx genes following a previously reported method^33^. Quantification of the expression levels of miR-122, TGFβ1 and TGFβR1 in these tissue samples revealed an eightfold decrease in miR-122 expression and an eightfold increase in the TGFβR1 level in tumour samples (Fig. 6a,b). However, the expression of TGFβ1 remained unchanged in these tissue samples. Although we observed a moderate increase in the number of blood vessels in the tumours (Fig. 6c), no metastatic nodules were found in the lung at 18 months in these HBx gene knock-in transgenic mice. To further examine these results, we obtained five mice with DEN-induced hepatocellular carcinoma. Quantitative analysis of miR-122 expression showed that it decreased ∼40% in the cancer samples (Supplementary Fig. 6f). In accord with this, a 15-fold increase in the TGFβR1 level was detected in the cancer samples, whereas TGFβ1 expression remained unchanged (Supplementary Fig. 6g). Taken together, these results demonstrated that miR-122 repression resulted in different patterns of pathological liver function in humans and mice *in vivo*, including tumour weight as well as angiogenesis and metastasis.

### miRNA switching targets is common in humans and mice

**Figure 7:**
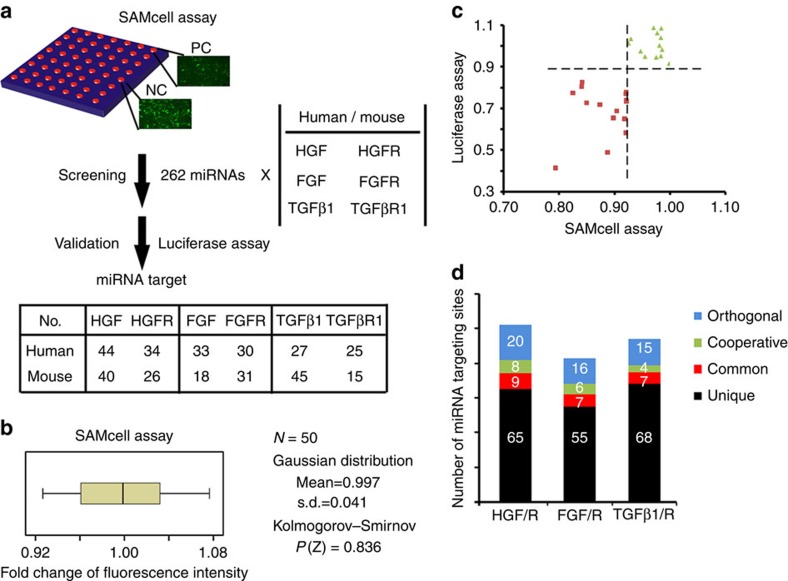
Genome-wide screening of miRNA targets in humans or mice. (**a**) Schematic diagram showing the screening strategy used to investigate the miRNA target sites along the 3′UTR of three pairs of ligands/receptors. (**b**) Cutoff value was set according to 50 control experiments. (**c**) SAMcell assay demonstrated the consistent results in the luciferase assay. Eight randomly selected miRNAs, including miR26a, 222, 365, 99b*, 496, 369, 99a or 216, were examined to target *HGF/HGFR* in humans and mice. (**d**) Column diagram showing the number of four types of miRNA target sites in *HGF/HGFR*, *TGFβ1/TGFβR1* or *FGF/FGFR*, including unique, common, cooperative and orthogonal type. Cooperative type refers to the situation in which a pair of a ligand and receptor was simultaneously targeted by one certain miRNA in one species.

To investigate whether this type of species-dependent miRNA signalling is specific to *TGFβ1/TGFβR1*, we performed a genome-wide screening of the miRNA-targeting sites along the 3′UTRs of three ligand/receptor pairs using a self-assembled cell microarray (SAMcell) we had previously developed^10^ (Fig. 7a). SAMcell allows the delivery of a large number of individual miRNA molecules to cell islands grown on single glass slides with a high level of efficiency and accuracy (Supplementary Fig. 7a). In brief, Hela cells stably expressing enhanced GFP (eGFP) fused with the 3′UTR from each gene were generated to reduce the variation from the plasmid transfection (Supplementary Fig. 7a). In case of targeting *CAT-1*, miR-122 was used as a positive control while let-7a as a negative control^34^. We synthesized 262 miRNAs with conserved sequences in humans and mice (Supplementary Table 3). The cutoff value was set to 0.92–1.08 on the basis of the Kolmogorov–Smirnov *Z*-test in 50 control experiments (*P*(*Z*)=0.836) (Fig. 7b). To assess the quality of our screening method, we randomly selected eight candidate miRNAs and validated the false positives using the conventional luciferase assays. All the tested miRNAs demonstrated consistent results in the luciferase assay, indicating excellent reproducibility of SAMcell assay (Fig. 7c; Supplementary Fig. 7b).

Noted, each gene was found to have more than tens of miRNA target sites (Fig. 7a; Supplementary Tables 4–6). In most cases, however, a given gene has a large proportion of unique miRNA target sites (Fig. 7d). For example, in the case of *HGF* or *HGFR*, 65 miRNAs target only one of them in humans and mice (or ‘species-unique' miRNA). In comparison with common miRNAs that target the same gene in both humans and mice, more than two times of the miRNAs were found to orthogonally regulate *HGF/HGFR*, *TGFβ1/TGFβR1* or *FGF/FGFR* across species, respectively. In addition, a few of the miRNAs simultaneously regulated both the ligands and receptors in a single species, indicating that these miRNAs may exert cooperative effects on a group of ligands/receptors.

## Discussion

In this study, we demonstrate that miR-122 targets different components in the TGFβ pathway, namely, *TGFβ1* in humans and *TGFβR1* in mice, thus providing the first evidence for species-dependent miRNA targeting within a pathway. This species-dependent miRNA regulation of ligand /receptor activity, convergent to the same signalling pathway, provides new perspective on the functional conservation of miRNAs. Under normal conditions, the inhibition of either the ligand or the receptor in the presence of miRNA results in a similar regulatory effect on downstream signalling molecules. However, this type of regulation might have markedly different consequences across species in the case of miRNA dysregulation, such as occurs in pathological and/or stressful conditions. Here our results clearly prove that a loss of miR-122 exerts markedly different effects on metastatic liver cancer in humans and mice.

In addition to cancer, the downregulation of miR-122 has been reported in many types of liver disease^35^. For example, the miR-122 level was decreased by 40% in subjects with nonalcoholic steatohepatitis or by 50–60% in patients with HCV infection compared with healthy controls. This finding presented here suggests potential new therapeutic or prophylactic strategies against liver and associated diseases. This study is of immediate relevance for a phase II clinical investigation Santaris Pharma recently performed. They used the anti-miRNA-122 agent miravirsen to treat HCV and reported no dose-limiting adverse events for <1 month. Our results indicate that the concentration of TGFβ1 needs to be carefully monitored in future clinical studies.

Furthermore, TGFβ1 is involved in immunosuppression^21^, heart disease^36^,^37^ and metabolic syndrome^38^, as well as other pathological conditions. It is highly possible that the imbalance of TGFβ1 due to the dysregulation of miR-122 makes a direct contribution to the development of these diseases. Indeed, there are some reports linking these diseases with liver abnormalities or HCC. For example, the patients with nonalcoholic fatty liver disease are at an increased risk of cardiovascular disease^39^,^40^, while the presence of metabolic abnormalities is significantly associated with the risk of HCC^38^,^41^.

Normally, TGFβ1 is a potent cytokine that plays a critical role in driving developmental programmes and controlling cell behaviour, including cell proliferation, differentiation, migration, immunosurveillance, tissue homeostasis and regeneration^42^. Mechanistically, the effects of TGFβ are different, or even opposite, depending on the cell types and conditions employed^42^. We believe that the addition of the liver-specific inhibition of TGFβ1 in humans and other primates may provide a unique context for liver development or function. After all, human liver demonstrates the unique physiology from mouse one. Murine cells do not permit the viral entry and inefficiently replicate HCV RNA^43^, and totally different transcription factors were required to convert fibroblasts into hepatocytes in humans or mice^44^,^45^. Undoubtedly, a deeper understanding of the physiological or pathological significance of this species-dependent miR-122 signalling lies in the investigation of monkeys with germline or conditional deletion of *Mir 122* in the future.

Finally, we performed a genome-wide screening of the miRNA target sites in the 3′UTRs of a group of ligands/receptors using the SAMcell assay. Over 50 miRNAs orthogonally targeting three pairs of ligands/receptors in a species-dependent manner were identified, thus indicating an evolutionarily common event. This finding is becoming markedly important when rethinking the paradox related to miRNA functions. For example, the genetic knockout of individual miRNAs in mice were reported to have not resulted in any obvious phenotypic difference in most cases^46^. In contrast, miRNA globally deregulates in human carcinoma and actively participates in the regulation of tumour development^5^,^47^. Together with another finding of a large proportion of ‘species-unique' miRNAs, we conclude that the knowledge of a miRNA obtained on basis of a mouse model might not be accurately applied to one in humans. In particular, some miRNAs identified here are tissue specific, such as the muscle-specific miRNA miR-208 targeting *FGF/FGFR*, the neuronal-rich miRNA miR-125b targeting *FGF/FGFR* and so on^2^,^48^,^49^. These tissue miRNAs are highly expressed, such as miR-122 outnumbering any single mRNA target by as much as 500-fold^50^. It is evident that the effect of a selective constraint of these tissue-specific miRNAs on ubiquitously expressing ligands/receptors might provide an easy and robust strategy controlling the protein level in specific cells in a specific species. Noted, the SAMcell assay is not limited to the identification of miRNA target sites in 3′UTRs. We already adapted this assay to characterize the miRNA target sites in the 5′UTRs and CDS through constructing the corresponding reporters, similar to ones shown in Fig. 3a,d (unpublished data). More cases of species-dependent miRNA targeting within a pathway would be identified. Thus, the biological outs of these miRNAs needed to be re-examined in species-dependent and global context. In sum, this study reveals a new mechanism for miRNA-mediated gene regulation underlying species-specific physiological or pathological phenotype and provides a novel insight for the understanding of miRNA biology across species, especially in humans.

## Methods

### siRNA duplex and miRNA mimics

siRNA duplexes and all human miRNA mimics were obtained from the GenePharma (Shanghai, China). The silence efficiency of these RNA interferenceconstructs for their targeted mRNAs was tested by quantitative real-time PCR at 24–48 h after transfection. All reactions were run in triplicate. All the primer sequences for cloning indicated genes were listed in Supplementary Table 7.

### Cell culture and transfection and infection

H22 was donated from Institute of liver diseases, Peking University People's Hospital, China, and HUVEC was donated from Professor Yanyi Huang at Peking University, China. Hela, HepG2, Huh7, 293T, 7721, LM9, ANC-1, MCF-7, Hepa1-6, NCTC1469 or LLC-PK1 cells were obtained from the Cell Resource Center, Peking Union Medical College (which is the headquarter of National Infrastructure of Cell Line Resource). All the cell lines were checked free of mycoplasma contamination by PCR and culture. Its species origin was confirmed with PCR. The identity of the cell line was authenticated with short tandem repeat (STR) profiling (FBI, CODIS).

Hela, HepG2, Huh7, 293 T, 7721, LM9, PANC-1, MCF-7, Hepa1-6, H22, NCTC1469 and C5.18 cells were cultured in high-glucose DMEM containing 10% fetal bovine serum (FBS), 100 U ml^−1^ penicillin and 0.1 mg ml^−1^ streptomycin, while low-glucose DMEM was used when culturing NIT-1 cells, and RPMI-1640 was used when culturing H22 cells. NCTC 1469 cells were cultured in DMEM containing 10% horse serum, 100 U ml^−1^ penicillin and 0.1 mg ml^−1^ streptomycin. HUVECs, which are donated from Professor Yanyi Huang at Peking University, China, were cultured for three to four passages in endothelial cell medium (EMC) medium (ScienCell) containing 1% endothelial cell growth supplements (ECGS), 5% FBS and 1% P/S in mouse-tail collagen-coated culture dishes. Pig kidney epithelial cells (LLC-PK1) were cultured in M199 medium (Gibco) containing 3% FBS and 1% P/S. All the cells were cultured under humidified conditions in 5% CO_2_ at 37 °C. Cells were washed with PBS and incubated in 0.25% trypsin containing 5 mM EDTA every 2–3 days. Medium containing corresponding concentration of FBS were added to terminate trypsin. After centrifugation, cells were diluted by the medium, counted via hemocytometer and then planted with appropriate concentration.

To achieve transient expression, plasmids were transfected using Lipofectamine 2000 (Invitrogen). The cell number and nucleotide amount followed the manufacturer's protocol. To establish stable cell lines, the indicated lentiviral vector was packaged and transfected into cells. In brief, lentivirus was packaged in 293T cells, and then infected to HepG2 or SMMC-7721 cells. After 72 h, eGFP-positive cells were collected by fluorescence-activated cell sorting. Alternatively, 48 h after infection, medium was changed, and puromycin (Sigma) was added to the medium with a final concentration of 2 μg ml^−1^. The selection was carried out for 3–5 weeks, and the stable cell lines of HepG2 were referred to as HepG2-NC, HepG2-122 (overexpressing miR-122) and HepG2-122-TGF (overexpressing miR-122 as well as TGF-β1), respectively.

### AGO-IP assay

The AGO-IP assay was performed following the previous description^23^. In brief, the lysate was cleared at 14,000*g* at 4 °C for 15 min and incubated with streptavidin beads for 30 min at 4 °C with gentle agitation. Beads were washed once with NP40 lysis buffer (50 mM HEPES pH 7.5, 150 mM KCl, 0.5% IGEPAL, 0.5 mM dithiothreitol (DTT), 2 mM EDTA, 50 U ml^−1^ RNAsin), treated with DNAse (PCR grade, Roche) for 15 min at room temeperature and washed five times with 1 ml of high-salt buffer (B&W buffer). Fifty microlitres of Protein G Dynabeads (Life Technologies) per 10-cm dish were washed twice with 1 ml of 1 × PBS. Added AGO2 antibody (Abcam, cat. # Ab32381; 4 μg; CST) diluted in 200 μl PBS with Tween-20, and incubated beads in room temperature for 10 min. Then, the beads were washed three times with 1 ml of NP40 lysis buffer and blocked for 1 h at 4 °C with 1 ml of NP40 lysis buffer containing bovine serum albumin (10 μg ml^−1^). Cell lysate was incubated with Ago2 antibody-coupled Protein G beads for 1 h at 4 °C with gentle rolling. The beads were washed five times with each 1 ml of immunoprecipitation wash buffer (50 mM HEPES, pH 7.5, 300 mM KCl, 0.05% NP40, 0.5 mM DTT and complete protease inhibitor (Roche)). After the last washing step, 200 μl digest buffer (100 mM Tris-HCl, pH 7.5, 150 mM NaCl and 12.5 mM EDTA) containing 240–440 μg proteinase K (recombinant PCR grade solution, Roche, Rotkreuz, Switzerland) was added to each sample and digested at 65 °C for 15 min. RNA was isolated by TRNzol (Tiangen) for reverse PCR and qPCR.

### Luciferase assays

For luciferase assay, the 5′UTR or 3′UTR of different genes were cloned into pGL3 plasmids, located at the 5′ or 3′ to the firefly luciferase gene, respectively. If not otherwise specified, 4.0 × 10^4^ Hela cells were co-transfected with 200 ng of the indicated pGL3 firefly luciferase construct and 20 ng of a pGL3 Renilla luciferase normalization control. In the meantime, the indicated miRNA expression plasmid or mimics was transfected. After 48 h, cells were lysed and luciferase activities were measured using the Dual Luciferase Reporter Assay System (Promega).

### Screening of the miRNA-targeting sites using SAMcell

The fabrication of the SAMCell microarray has been previously described^10^. In brief, the glass slides (2.2 × 2.2 cm) were covered with poly (*N*-isopropylacrylamide) (Aldrich) dissolved in ethanol (6% (w/v)). The slides were etched via a shadow mask by oxygen plasma for 3.5 min at 200 W power. The reverse transfection protocol refers to previous description^51^. The miRNA mimics library mixed in the reverse transfection reagent was printed on the chip (Suzhou Genoarray Co., Ltd.). Next, the slides were fixed in a six-well plate by melted wax. We built a reporter system that expressed a Venus protein, and then the 3′UTRs of different genes from human or mouse were cloned as 3′UTRs of Venus (showed in supplementary Fig. 7a). A stable Hela cell line expressing a reporter system was selected by use of fluorescence-activated cell sorting as described above. Over 260 miRNAs were printed on SAMcell microarrays. Then, 5 × 10^5^ cells containing 3′UTR reporter were transferred in each well. About 24–48 h later, the dishes were moved at room temperature for 5 min and washed with PBS for three times to ensure the total removal of the polymer. Average fluorescent intensity of each cell island were collected and analysed. The cutoff value was obtained on the basis of the Kolmogorov–Smirnov *Z*-test in 50 control experiments. For each miRNA, at least six times were repeated, and the statistical analysis was shown in Supplementary Tables 4 and 5.

### Human liver tissues and miRNA measurement

Human primary liver cancer tissues and normal adjacent tissue samples were obtained from Peking University Third Hospital. Before surgery, all patients provided written informed consent to allow any excess tissue to be used for research studies. According to the American Joint Committee on Cancer 6th edition TNM classification, patients were classified as stage I, II or III. Immediately after surgical resection, all tissues were snap-frozen and stored in liquid nitrogen. Quantitative examination of miRNA expression in cell lines and tissue samples followed the method previously established^52^.

### Supernatant collection and ELISA

Different cells were planted with appropriate concentrations, and after 48 h cells would reached ∼80% confluence. The medium was then moved into sterilized tubes. After centrifugation, the supernatant was collected and stored at −80 °C. For neutralizing assay, the supernatant was preincubated with 1 μg ml^−1^ neutralizing antibody (R&D) for 2 h at room temperature before centrifugation. The concentration of TGFβ1 and VEGF in the supernatant was determined by ELISA. The TGFβ1 Emax ImmunoAssay System was purchased from Promega, and the VEGF ELISA kits were purchased from R&D Systems. The assay was performed according to the manufacturer's protocol.

### Immunofluorescence

Huh7 cells (1 × 10^5^) were seeded per well in a six-well plate. After attachment, cells were FBS-starved overnight, and then the medium was changed with different supernatant as indicated. As a positive control, recombinant TGFβ1 (Sino Biological Inc.) was added to another well with a final concentration of 2.5 ng ml^−1^. After another 24 h, cells were fixed with 4% paraformaldehyde, permeabilized with 0.1% Triton X-100 and blocked in 2% bovine serum albumin for 1 h at room temperature. The expression of E-cadherin or vimentin was examined using their antibodies and visualized using anti-Rabbit or anti-Mouse IgG (H+L), F(ab′)2 Fragment (Alexa Fluor 488 Conjugate; Cell Signalling Tech.), respectively. All the fluorescent images were taken by Nikon TE2000-E (CCD: Regita 2000R, Qimaging, Canada).

### Immunoblotting

In brief, total protein extracted from either cell lines or tissues were resolved by SDS–polyacrylamide gel electrophoresis, and then transferred to a polyvinylidene difluoride membrane (Millipore Corporation). The membranes were probed with antibodies against TGFβ1 (R&D, cat. # MAB240), TGFβ2 (R&D, cat. # MAB612), TGFβ3 (R&D, cat. # MAB643), TGFβR1 (R&D, cat. # MAB5871), Smad2 (Cell Signaling Technology, cat. # 3103S), Phospho-Smad2 (Cell Signaling Technology, cat. # 3101S), E-cadherin (Cell Signaling Technology, cat. # 3195S), vimentin (Abcam, cat. # Ab8978) or β-actin (Santa Cruz, cat. # SC-7210). The images were obtained and quantified by Quantity One software (Bio-Rad). The final dilutions of primary and secondary antibodies were 1:1,000 or 1:3,000, respectively. Images have been cropped for presentation and full size images are presented in Supplementary Figs 9–18.

### Immunohistochemistry

Immunohistochemistry was accomplished at Chinese Academy of Medical Sciences. In brief, tissue samples were fixed in 4% paraformaldehyde over night at room temperature, followed by a wash with PBS and transferred to 70% ethanol, and then embedded in paraffin, sectioned and stained with haematoxylin and eosin. The immunohistochemistry detection with anti-CD31 antibodies (BD Biosciences) was performed on paraffin sections. The vessel number was counted by ImageJ software.

### *In vitro* matrigel angiogenesis assays

HUVECs (5 × 10^4^) were seeded per well in a 24-well plate coated by matrigel (BD Biosciences). Six hours later, the cells attached and the medium was changed with different supernatant mentioned in the article. Recombinant TGFβ1 (Sino Biological Inc.) was added to other wells with a final concentration of 2 ng ml^−1^. HUVECs cultured with fresh medium as blank. After another 24 h, images were got and tubular lengths and BPNs were measured and quantified by ImageJ software. In brief, the Matrigel angiogenesis was first photographed. When pictures were opened with imageJ, the software will classify targets into tubulin and cells by length–width ratio. Then, we counted the total length and branches of tubulin manually and analysis.

### Animal model and *in vivo* tumorigenesis assay

Mouse HCC models were constructed following previous reports^33^. For *in vivo* tumorigenesis assay, 1 × 10^7^ HepG2-NC, HepG2-122 and HepG2-122-TGF cells were subcutaneously implanted into each BALB/c Nude mouse (5-month-old males and females, Vitalriver, China), respectively. After 5 weeks, mice were killed and the tumours were weighed. All research involving animals complied with protocols approved by the Laboratory Animal Center, Peking University. For subcutaneous tumour implantation, HepG2 or Hepa1-6 cells were suspended in PBS and injected into the dorsum of mice. For tail vein tumour cells injection, HepG2 or Hepa1-6 cells were suspended in PBS and injected into the tail vein of mice, and after 5 weeks, the lungs and other tissues were obtained to examine metastatic nodules.

*In vitro* matrigel angiogenesis assays were carried out as previously described^34^. In brief, 24 h after infection of miR-23b sponge or NC, HUVECs were seeded on matrigel (BD Biosciences) in a 96-well plate (Sigma).Tube lengths and branches were measured and quantified by Image J software.

### Statistical analysis

For statistical analysis, two-sided Student's *t*-tests were processed by Excel spreadsheet. A *P* value <0.05 was considered statistically significant. *P<0.05; **P<0.01; ***P<0.001. Error bars represented s.d.'s of at least three independent experiments.

## Additional information

**How to cite this article:** Yin, S. *et al*. Differential TGFβ pathway targeting by miR-122 in humans and mice affects liver cancer metastasis. *Nat. Commun.* 7:11012 doi: 10.1038/ncomms11012 (2016).

## Supplementary Material

Supplementary Information Supplementary Figures 1-18, Supplementary Tables 1-7
