# Supplementary material for: Differential TGFβ pathway targeting by miR-122 in humans and mice affects liver cancer metastasis
Source: Nat Commun. 2016 Mar 18;7:11012. doi: 10.1038/ncomms11012 (PMC4802055; doi:10.1038/ncomms11012)
Supplement: Supplementary Information — Supplementary Figures 1-18, Supplementary Tables 1-7 [file ncomms11012-s1.pdf]

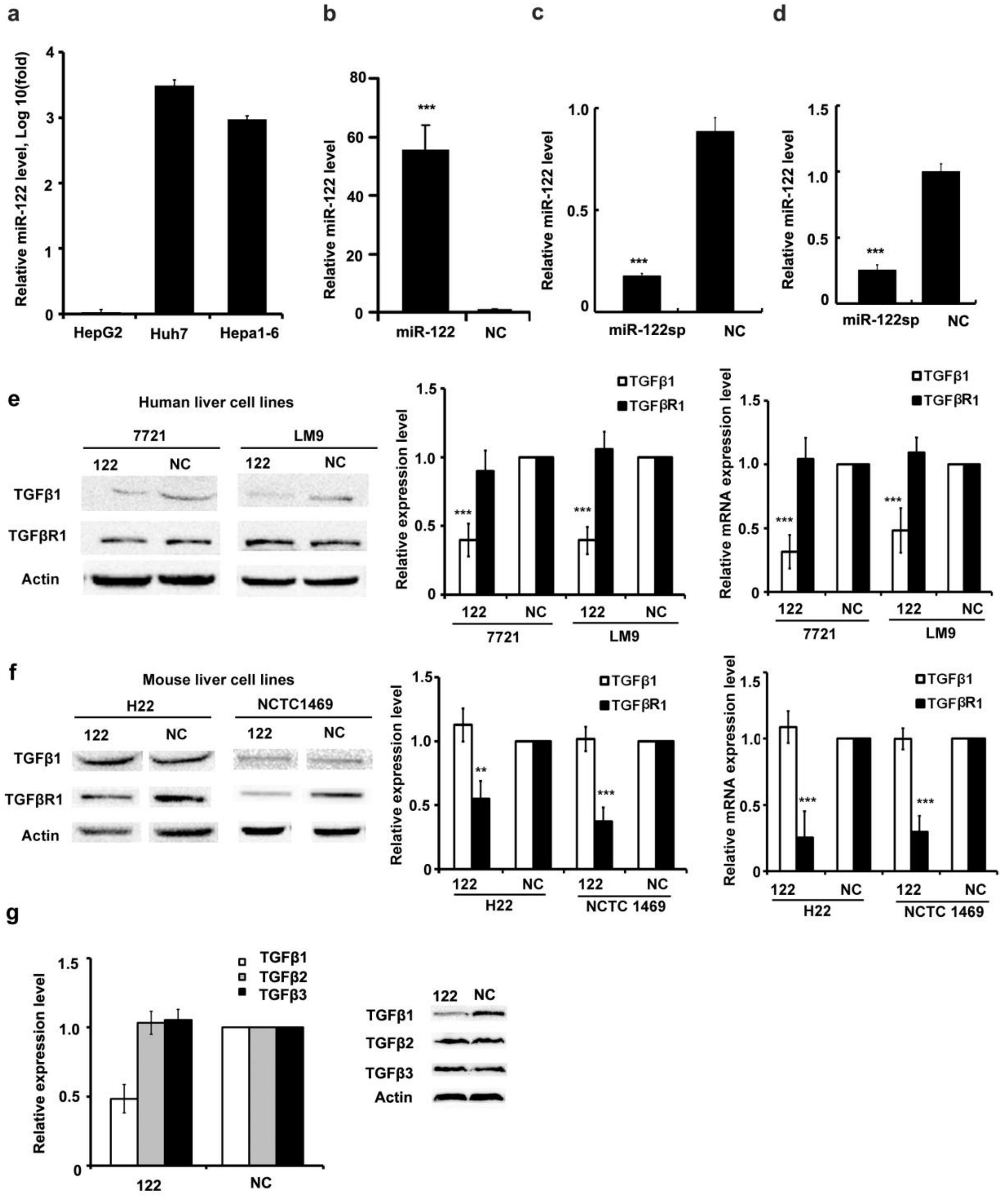

Supplementary Figure S1. Species-dependent targeting TGFβ1/TGFβR1 by miR-122

a. Expression of miR-122 in HepG2, Huh7 or Hepa1-6 cells. Three independent repeats are performed.

b. miR-122 level detected by the AGO-IP assay in transfected HepG2 cells. Three independent repeats are performed.

c. Expression of miR-122 in Huh7 cells transfected with miR-122 sponge and NC. Three independent repeats are performed in each experiment.

d. Expression of miR-122 in Hepa1-6 cells transfected with miR-122 sponge and NC. Three independent repeats are performed in each experiment.

e. Expression level of TGFβ1, TGFβ2 or TGFβ3 in HepG2-122 cells or HepG2 cells. Error bars, ± s.d * $P < 0.05$; ** $P < 0.01$; *** $P < 0.001$ by two-sided Student's *t* test. NC: negative control.

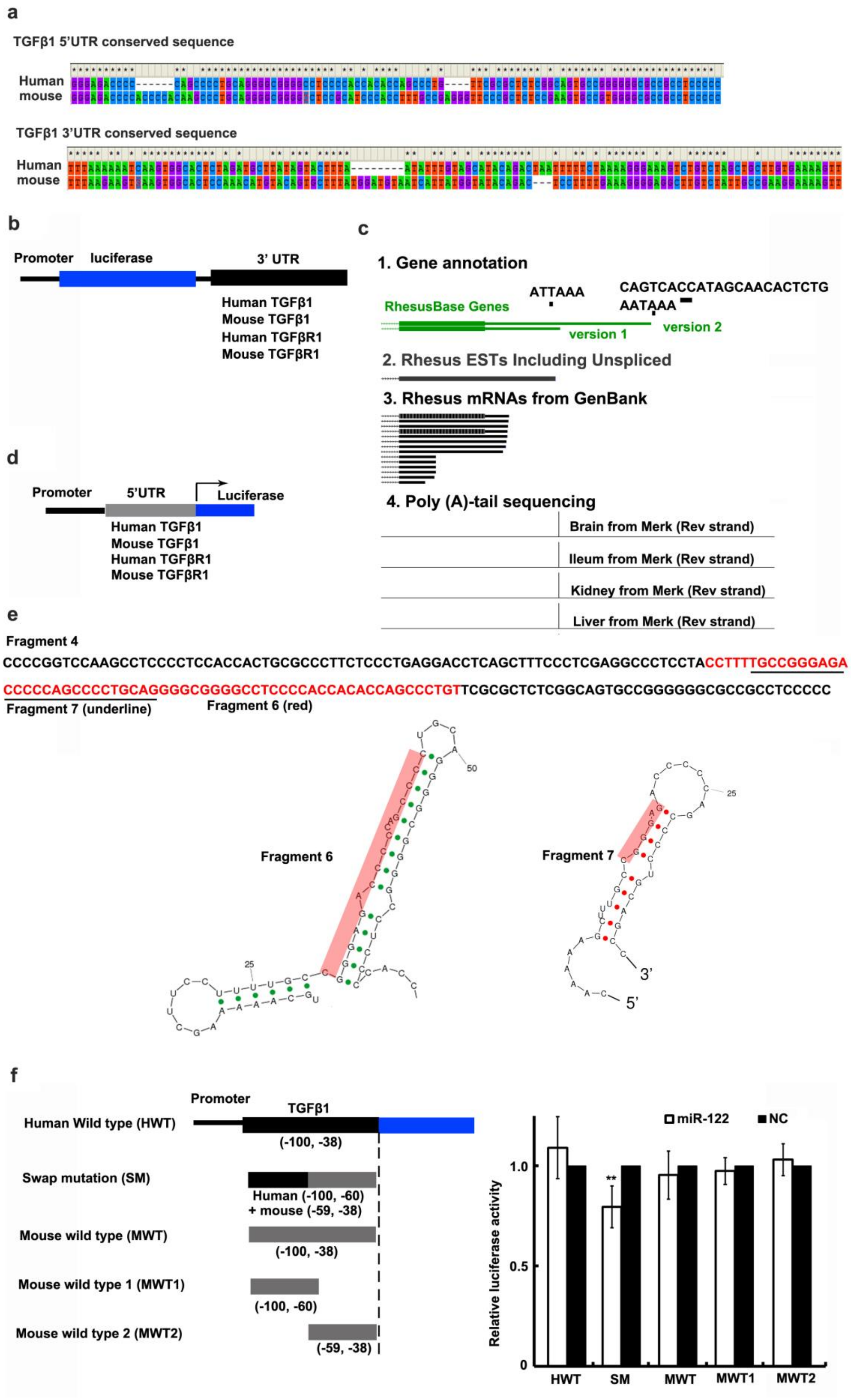
a
TGFβ1 5'UTR conserved sequence
Human
mouse
TGFβ1 3'UTR conserved sequence
Human
mouse
b
Promoter
luciferase
3' UTR
Human TGFβ1
Mouse TGFβ1
Human TGFβR1
Mouse TGFβR1
c
1. Gene annotation
ATTAAA
CAGTCACCATAGCAACACTCTG
AATAAA
RhesusBase Genes
version 1
version 2
2. Rhesus ESTs Including Unspliced
3. Rhesus mRNAs from GenBank
4. Poly (A)-tail sequencing
Brain from Merk (Rev strand)
Ileum from Merk (Rev strand)
Kidney from Merk (Rev strand)
Liver from Merk (Rev strand)
d
Promoter
5'UTR
Luciferase
Human TGFβ1
Mouse TGFβ1
Human TGFβR1
Mouse TGFβR1
e
Fragment 4
CCCCGGTCCAAGCCTCCCCTCCACCACTGCGCCCTTCTCCCTGAGGACCTCAGCTTTCCCTCGAGGCCCTCCTACCTTTTGCCGGGAGA
CCCCCAGCCCCTGCAGGGGCGGGGCCTCCCCACCACACCAGCCCTGTTCGCGCTCTCGGCAGTGCCGGGGGGCGCCGCCTCCCCC
Fragment 7 (underline)
Fragment 6 (red)
Fragment 6
Fragment 7
f
Promoter
TGFβ1
Human Wild type (HWT)
(-100, -38)
Swap mutation (SM)
Human (-100, -60)
+ mouse (-59, -38)
Mouse wild type (MWT)
(-100, -38)
Mouse wild type 1 (MWT1)
(-100, -60)
Mouse wild type 2 (MWT2)
(-59, -38)
miR-122
NC
Relative luciferase activity
1.0
0.5
0
**
HWT
SM
MWT
MWT1
MWT2

Supplementary Figure S2. The identification of the targeting site of miRNA-122 in TGFβ1 5'UTR

a. Comparative analysis of 5'UTR or 3'UTR conserved sequence in TGFβ1 or TGFβR1 in humans and mice.

b. Diagram showing a luciferase reporter construct containing different 3'UTRs.

c. Evidences showing the amount of different versions of Rhesus monkey TGFβ1 mRNAs, which includes gene annotation from UCSC Genes and ESTs, GenBank (NCBI), or PolyA sequencing data of different tissues (Derti et al., 2012).

d. Diagram showing a luciferase reporter construct containing different 5'UTRs.

e. Sequences of fragment 4, 6 (highlighted in red) or 7 (highlighted in underline) and predicted secondary RNA structure of fragment 6 or 7, in which the sequence targeted by miR-122 was shadowed in red.

f. Swap mutation experiments to identify the targeting region of miR-122 in human TGFβ1 5'UTR. Six independent repeats are performed in each experiment.

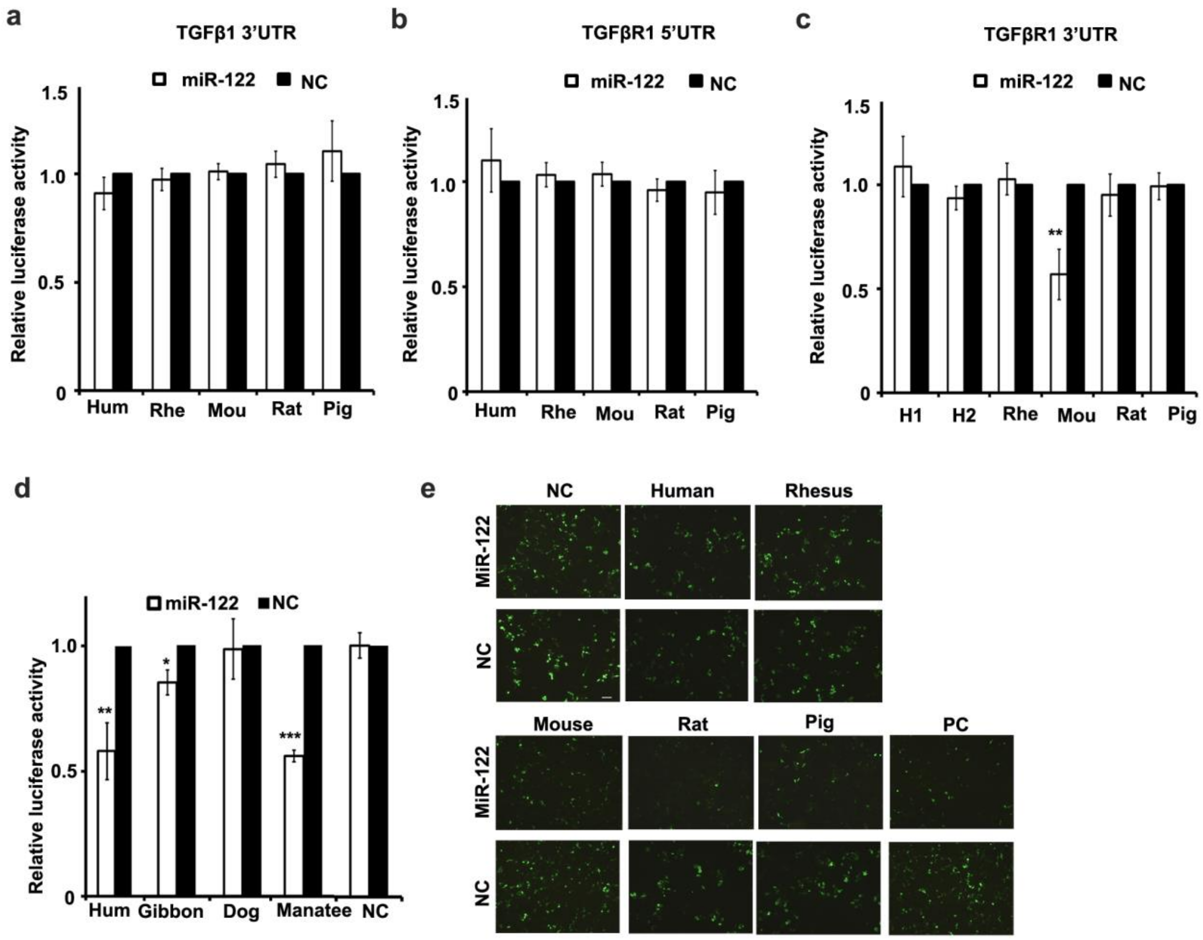

Supplementary Figure S3. Molecular mechanism of switch of miR-122 targeting of TGFβ1/ TGFβR1 across vertebrate species

a. Luciferase activity was measured after transfection of the indicated reporter constructs. TGFβ1 3'UTRs were cloned into 3'UTR of luciferase in a pGL plasmid, respectively. Hum: Human, Rhe: Rhesus monkey; Mou: Mouse. Six independent repeats are performed in each experiment.

b. Luciferase activity was measured after transfection of the indicated reporter constructs. TGFβR1 5'UTR was cloned into the promoter region. Hum: Human, Rhe: Rhesus monkey; Mou: Mouse. Six independent repeats are performed in each experiment.

c. Luciferase activity was measured after transfection of the indicated reporter constructs. TGFβR1 3'UTR was cloned into 3'UTR of luciferase in pGL plasmid. The 3'UTR of human TGFβ1 was divided into two parts, H1 or H2, because of its long

length. Six independent repeats are performed in each experiment.

d. Luciferase activity was measured after transfection of the indicated reporter constructs. The target sequence of TGFβ1 5'UTR in different species was cloned into the promoter region of pGL plasmid. Six independent repeats are performed in each experiment.

e. Fluorescent images showing Hela cells transfected with miR-122 as well as the indicated reporter constructs. All the candidate sequences were cloned in the CDS of a fusion protein of luciferase and eGFP. The construct of a reporter was shown in Figure 3C. Six independent repeats are performed in each experiment.

Error bars, ± SD * $P < 0.05$; ** $P < 0.01$; *** $P < 0.001$ by two-sided Student's *t* test. NC: Negative control.

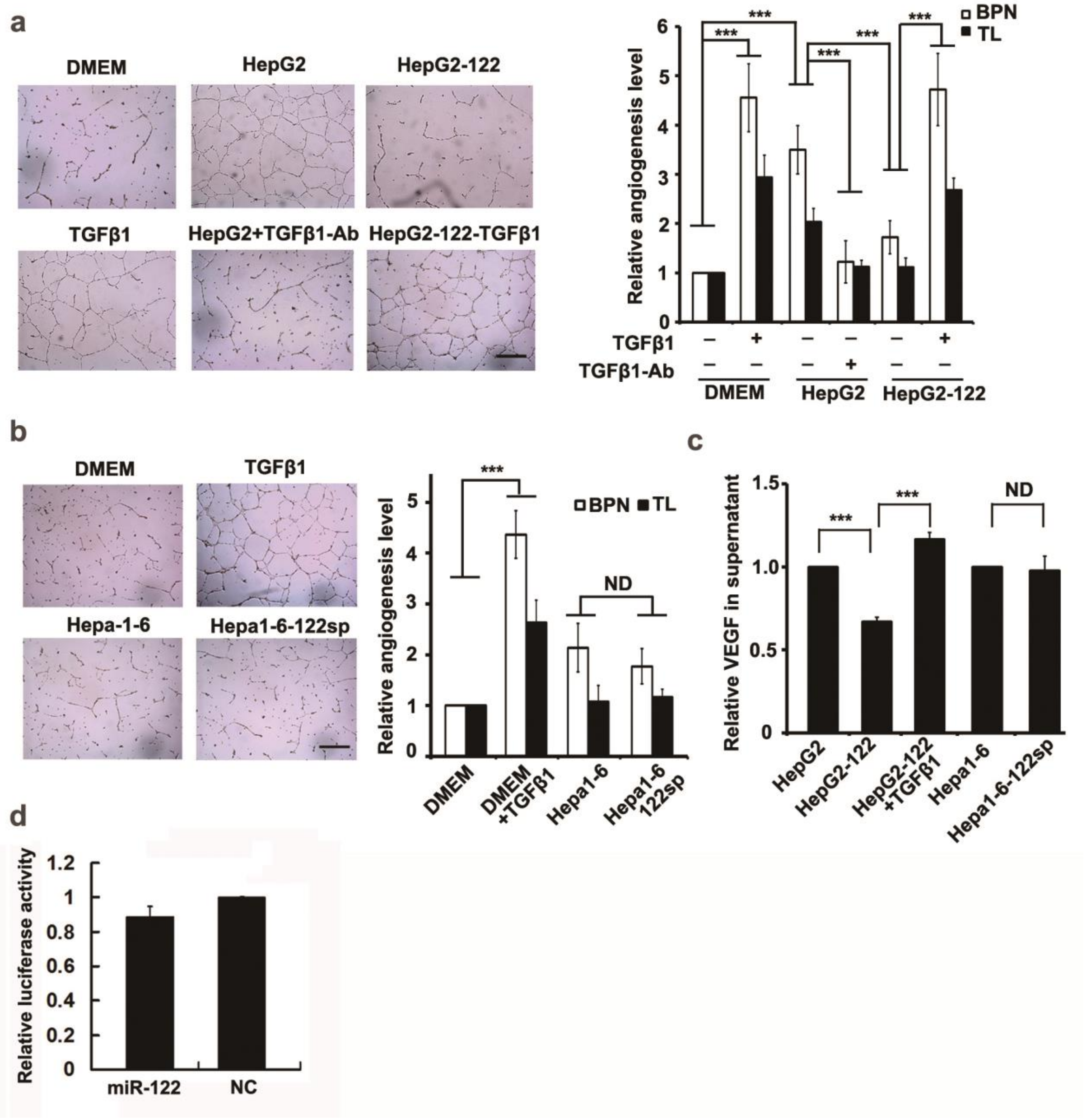

Supplementary Figure S4. MiR-122 has a distinct impact on angiogenesis in human or mouse cells

a, b Repressive images showing endothelial cells that were treated as indicated in 24 hours after planting. Quantitation analysis of the tubule branch point number (BPN) and tubule length (TL) is shown in right, respectively. Scale bar: 500 μm. Three independent repeats are performed in each experiment.

c. Expression level of VEGF in HepG2 or Hepa1-6 cells, treated as indicated, was quantified by ELISA assay. Three independent repeats are performed in each experiment.

d. Luciferase assay shows that miR-122 does not directly target VEGF. Six independent repeats are performed in each experiment.

Error bars, ± SD * $P < 0.05$; ** $P < 0.01$; *** $P < 0.001$ by two-sided Student's *t* test. ND: No difference.

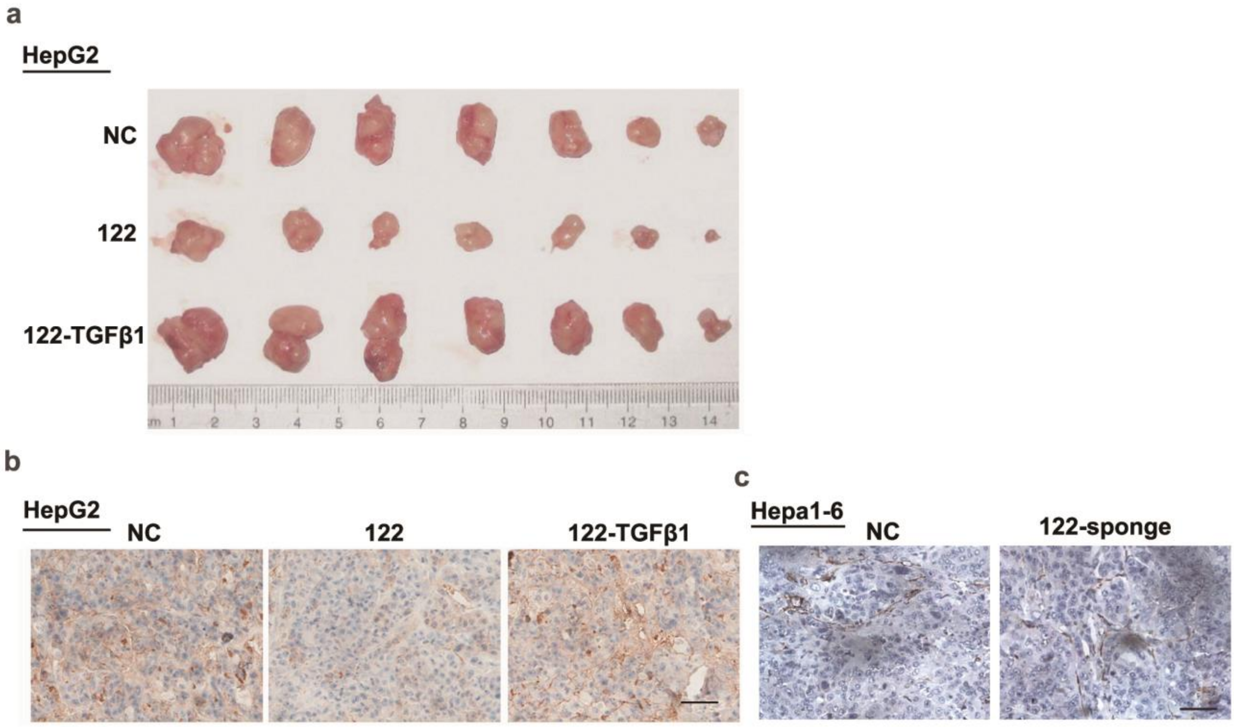

Supplementary Figure S5. Loss of miR-122 caused the different metastatic effects in human xenografts or mouse allografts

a. Images showing tumor size in mice implanted with the indicated cells. Seven independent repeats are performed in each experiment.

b, c. CD31 stained sections of liver cancer tissues as indicated. Seven independent repeats are performed in each experiment. Scale bar: 50 μm.

Error bars, ± s.d * $P < 0.05$; ** $P < 0.01$; *** $P < 0.001$ by two-sided Student's *t* test.

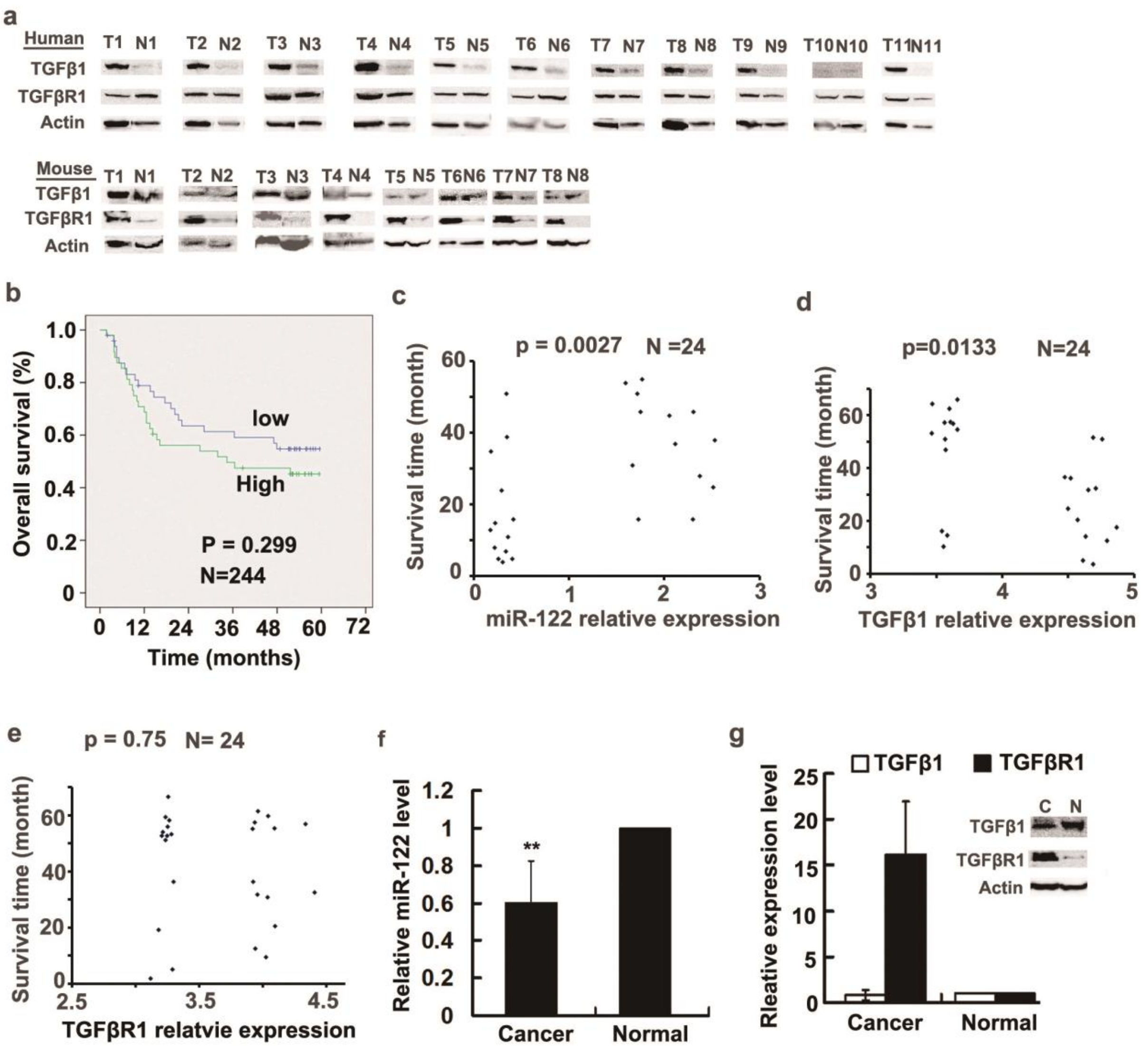

Supplementary Figure S6. Loss of miR-122 caused the different metastatic effects in human or mouse hepatocellular carcinoma samples

a. Western blot analysis of TGFβ1 or TGFβR1 in human or mouse liver cancer samples as well as normal adjacent samples. 11 pairs of human tissue samples and 8 pairs of mouse tissue samples were studied. Actin as loading control.

b. Kaplan–Meier curves for overall survival in HCC cohort from the Liver Cancer Institute (LCI) and Zhongshan Hospital. Expression value=log2 of RMA-calculated Signal Intensity. TGFβR1 expression value <4 as low expression group, while TGFβR1 expression value >5 as high expression group. n=244 patients; P-value based on the Mantel–Cox log-rank test.

c. Representative data of miR-122 expression were shown. N=24 patients.

d-e. Representative data ofTGF-β1 or TGF-βR1 expression were shown in 24 patients.

f. Quantitative analysis of miR-122 levels in hepatocellular tumors in tissues as indicated (eight independent repeats).

g. Western blot assay showing the expression levels of TGFβ1/TGFβR1 in tissues as indicated. Representative images are shown in the inset. Eight independent repeats are performed in each experiment. C: cancer; N: normal. Tβ:TGFβ1; TβR: TGFβR1. Error bars, ± s.d * $P < 0.05$; ** $P < 0.01$; *** $P < 0.001$ by two-sided Student's *t* test.

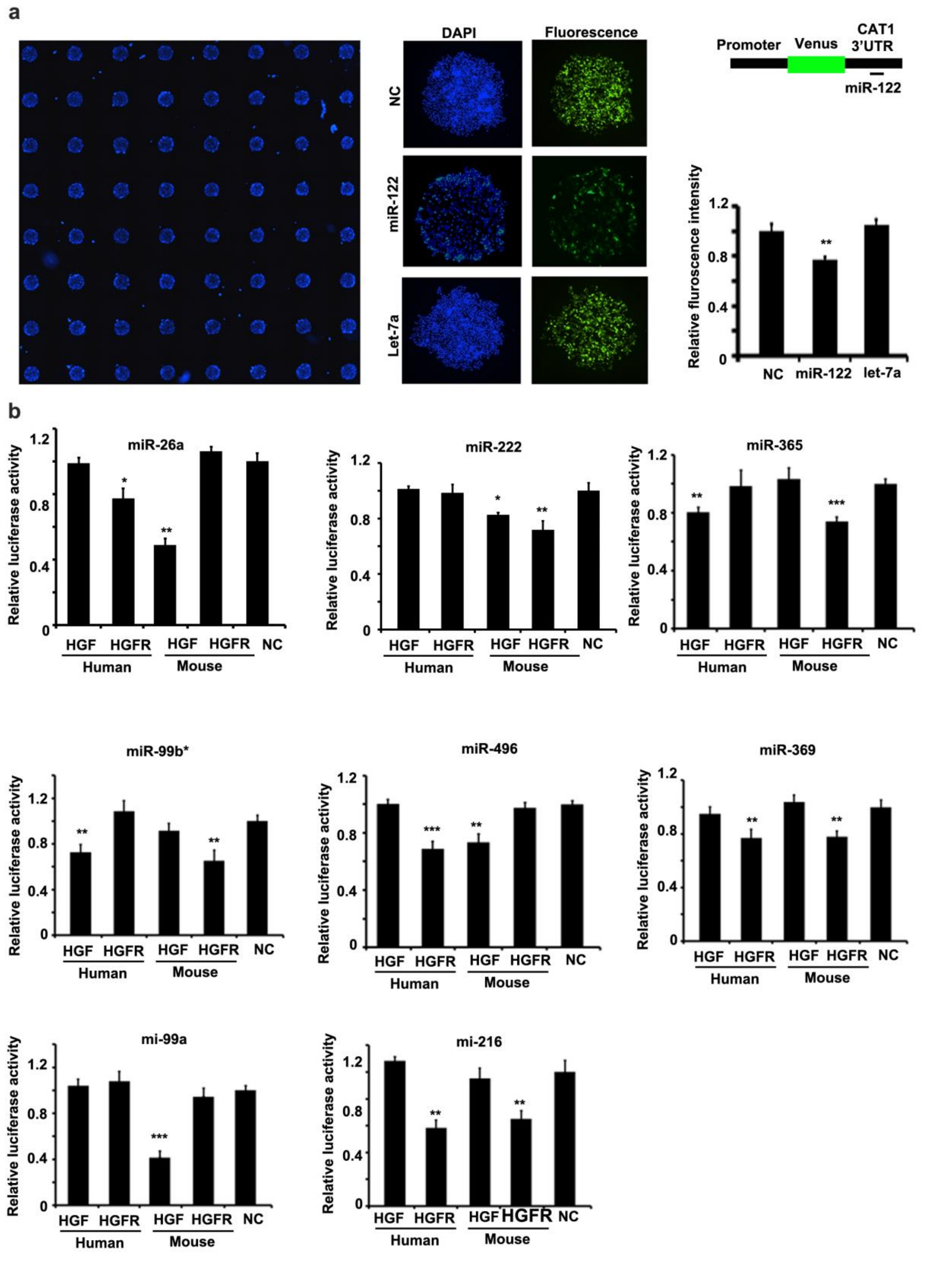

Supplementary Figure S7. Genome-wide identification of miRNA targeting sites along HGF/HGFR, TGFβ1/TGFβR1, or FGF/FGFR

a. SAM cell assays for the identification of miRNA targeting sites. (Left) an array of cell islands that cells were stained in DAPI was shown; (Middle, Right) Fluorescent signals

were quantified in cells expressing a Venus-3’ UTR (CAT1) construct as well as indicated miRNAs.

b. Validation of randomly selected hits obtained in (A) by use of luciferase assay. Six independent repeats are performed in each experiment.

Supplementary Figure S9. Western blot (Figure 1a)

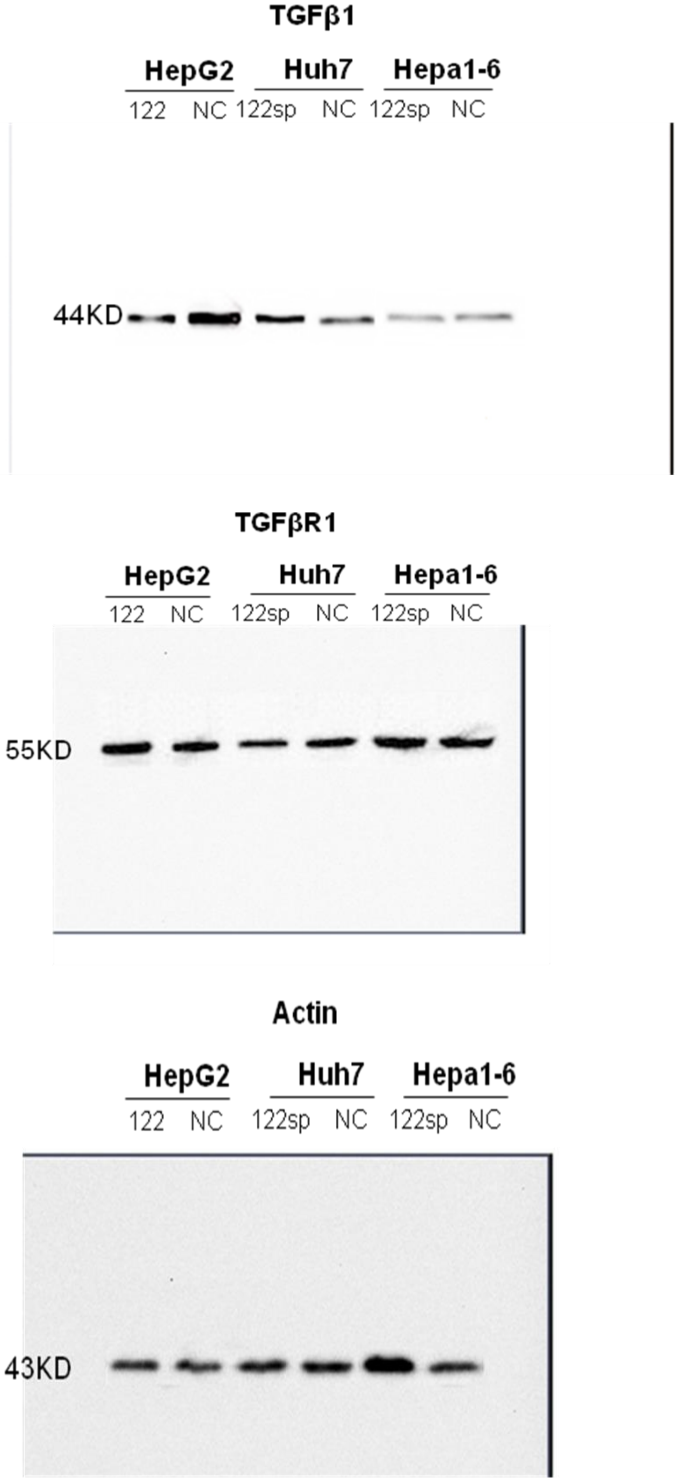

Supplementary Figure S10. Western blot (Figure 1c)

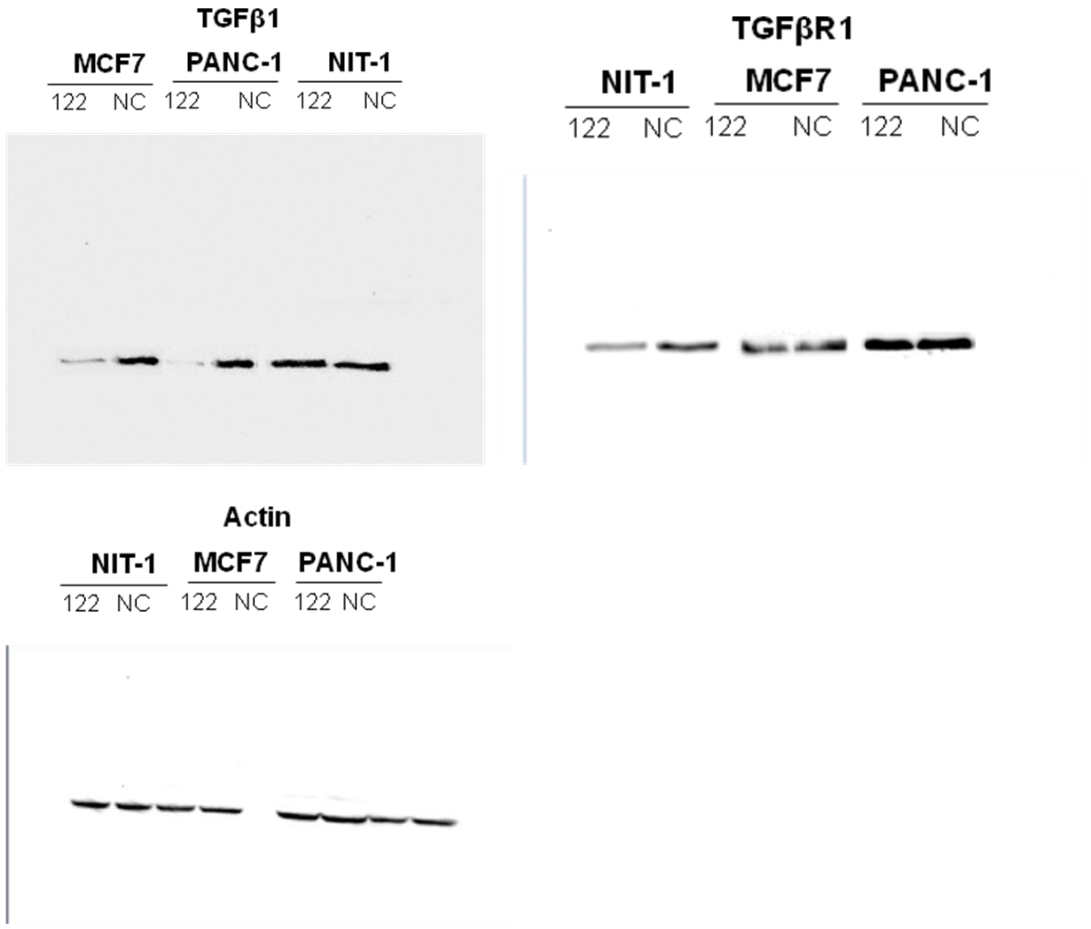

Supplementary Figure S11. Western blot (Figure 1e)

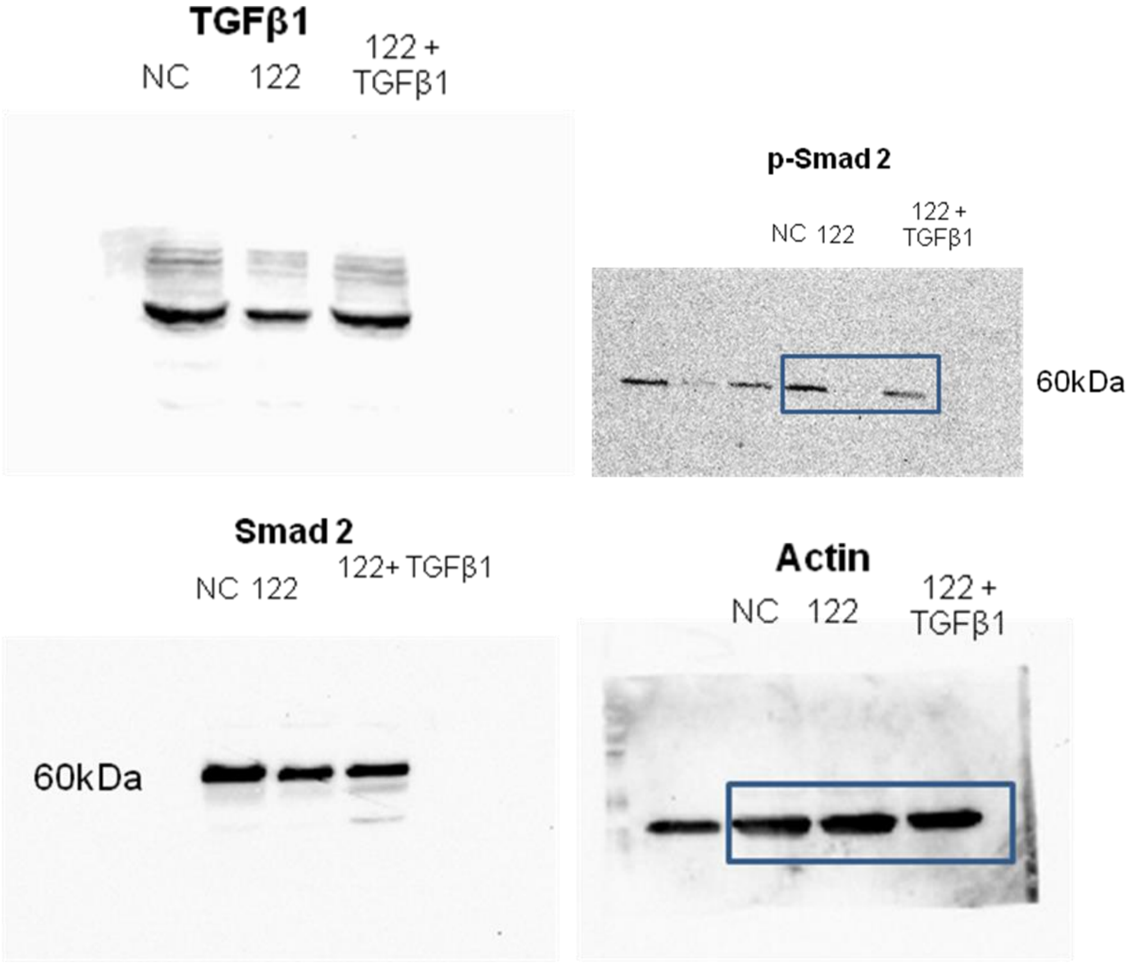

Supplementary Figure S12. Western blot (Figure 1f)

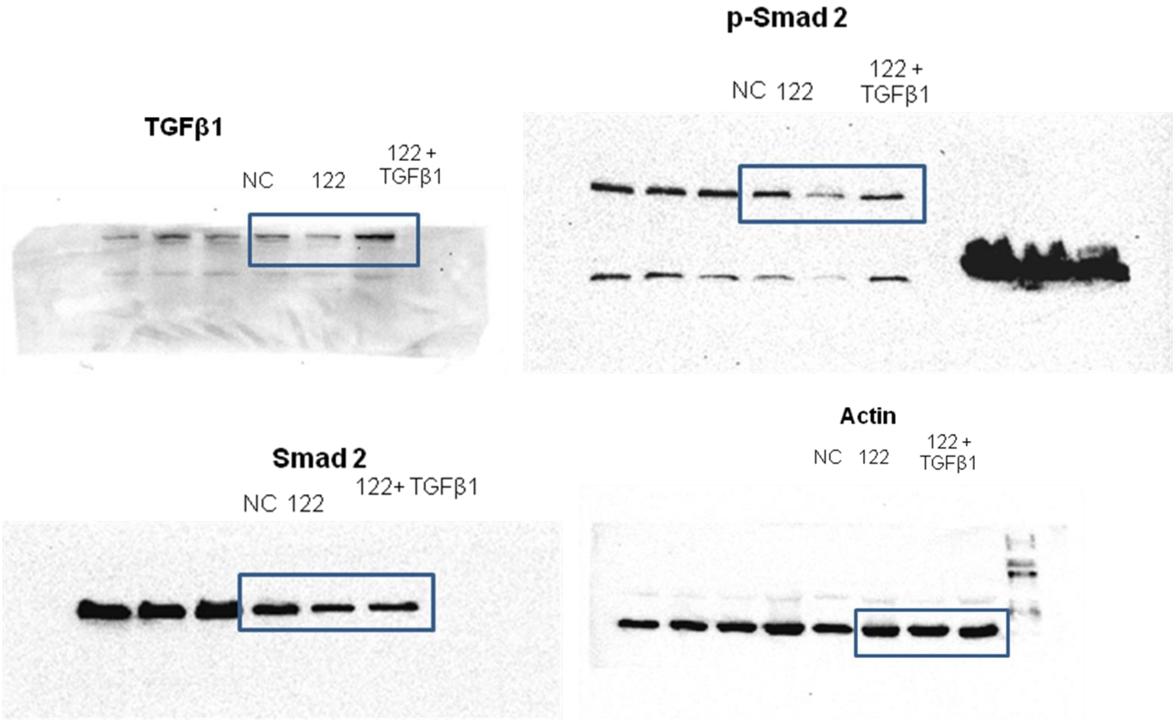

Supplementary Figure S13. Western blot (Figure 1g)

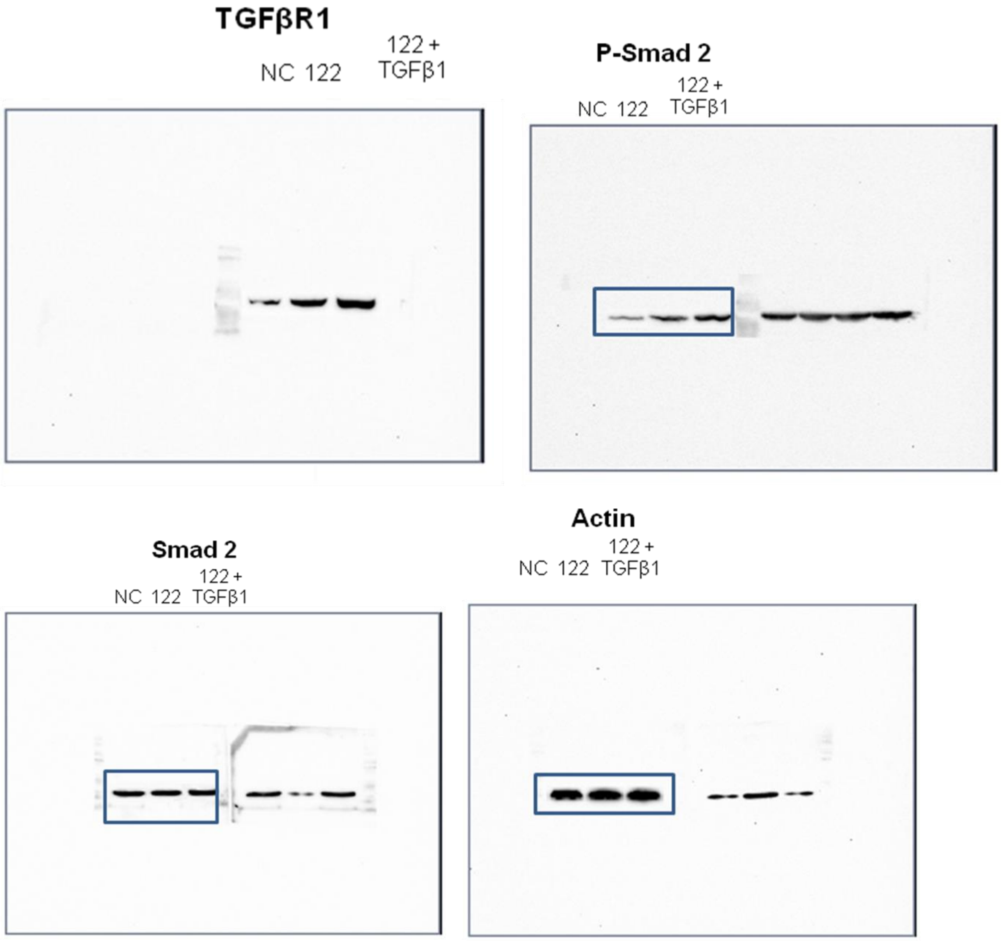

Supplementary Figure S14. Western blot (Figure 4a)

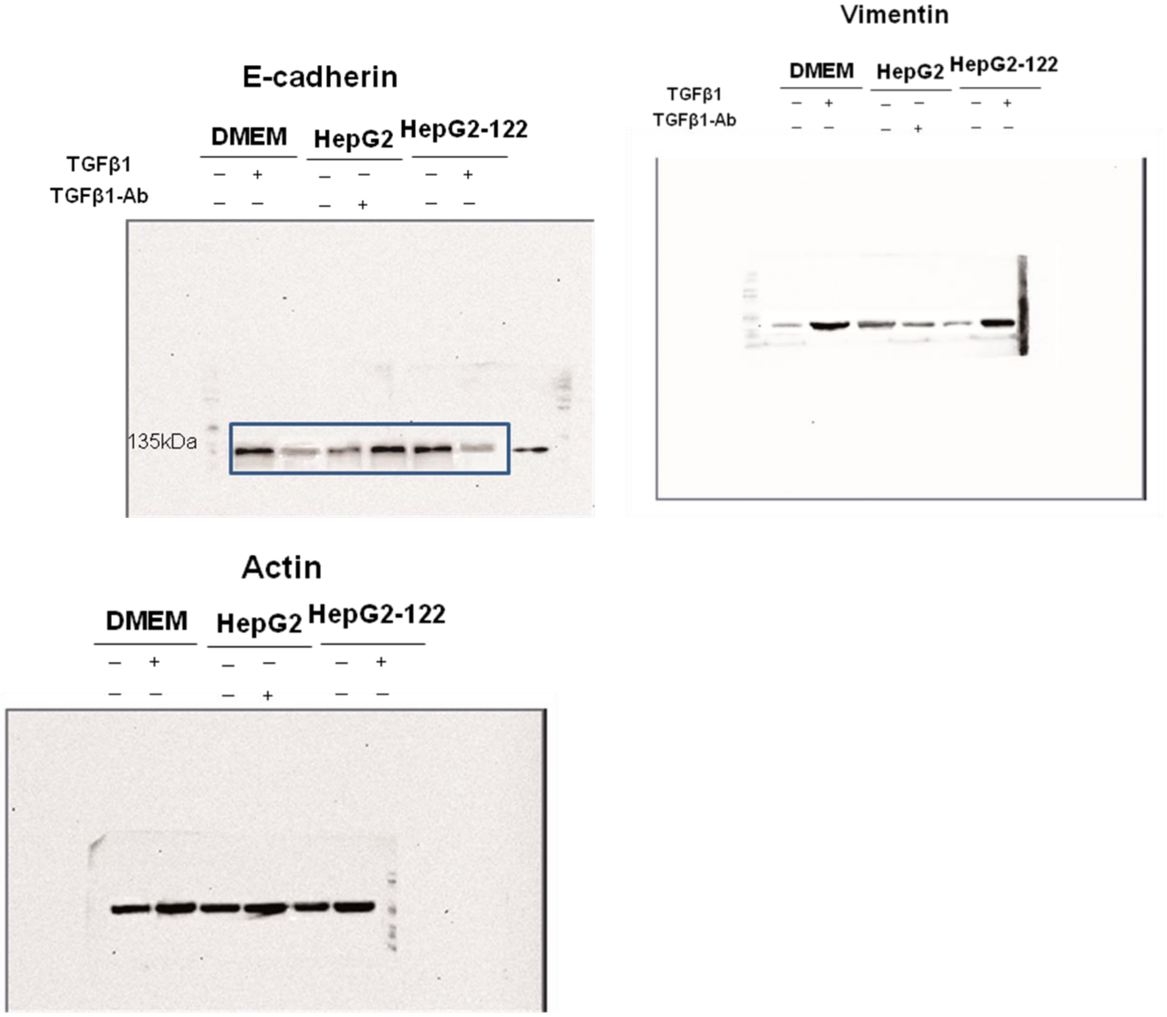

Supplementary Figure S15. Western blot (Figure 4f)

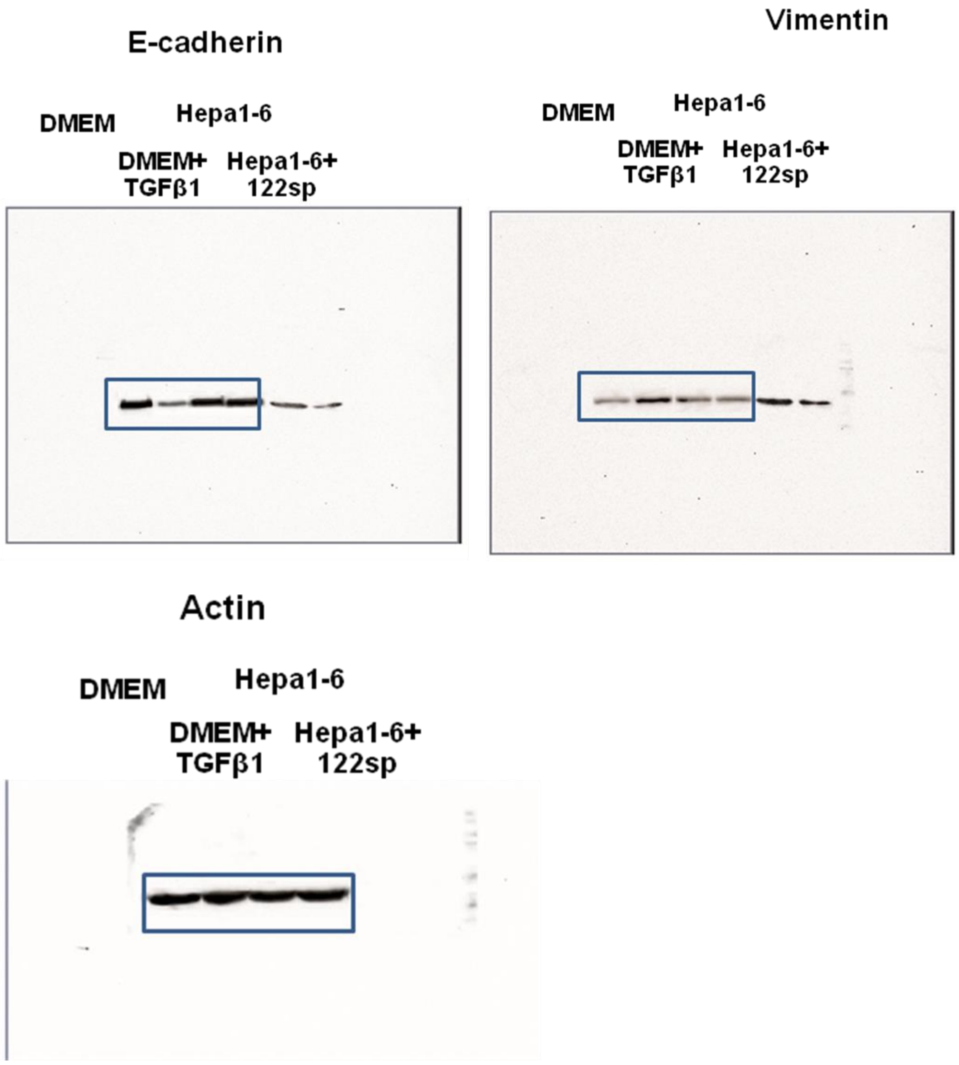

Supplementary Figure S16. Western blot (Figure s1e)

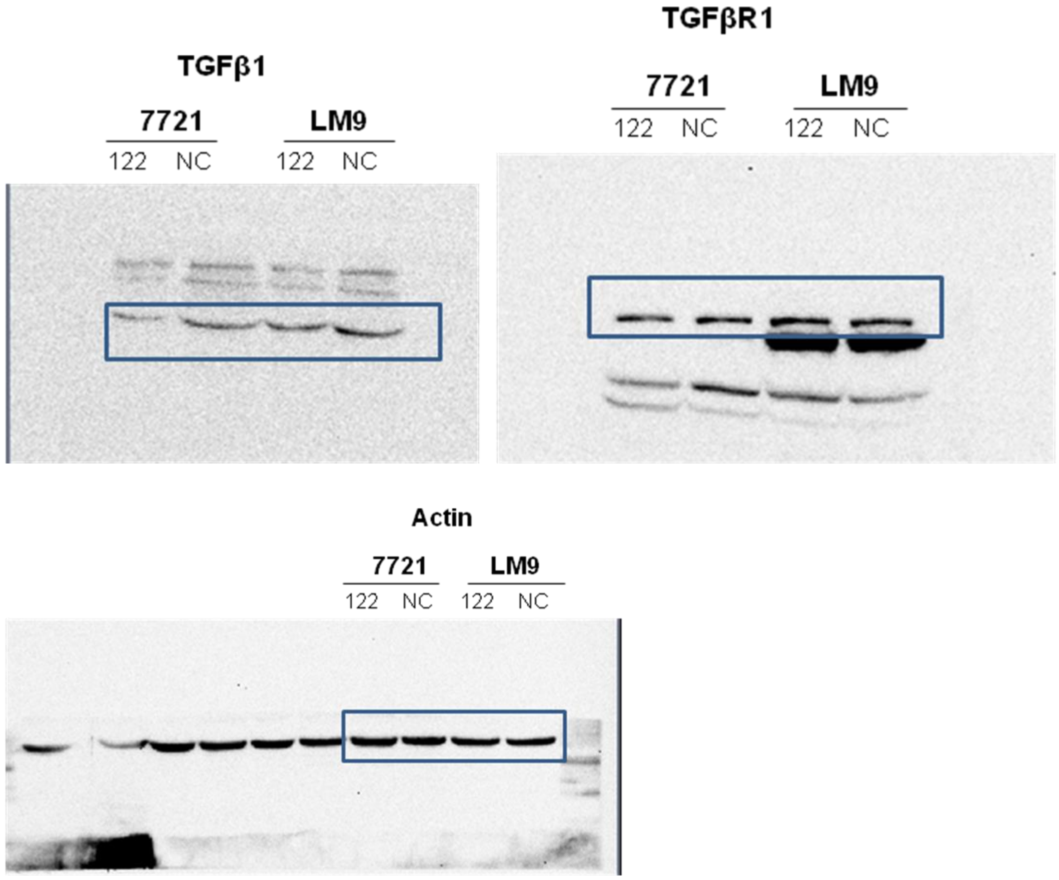

Supplementary Figure S17. Western blot (Figure s1f)

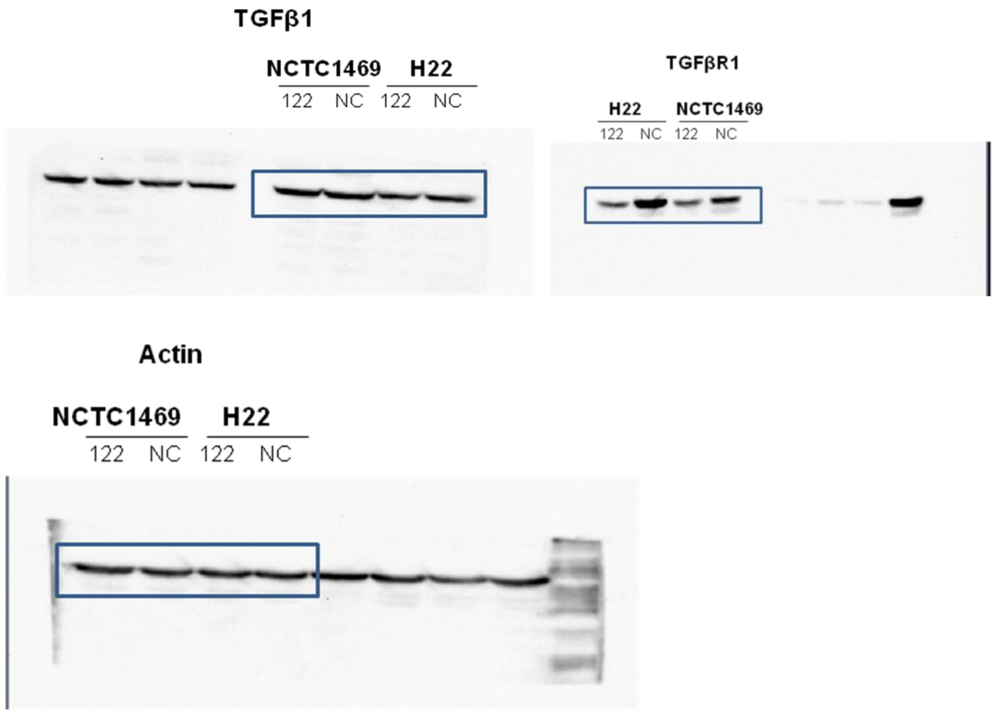

Supplementary Figure S18. Western blot (Figure s6)

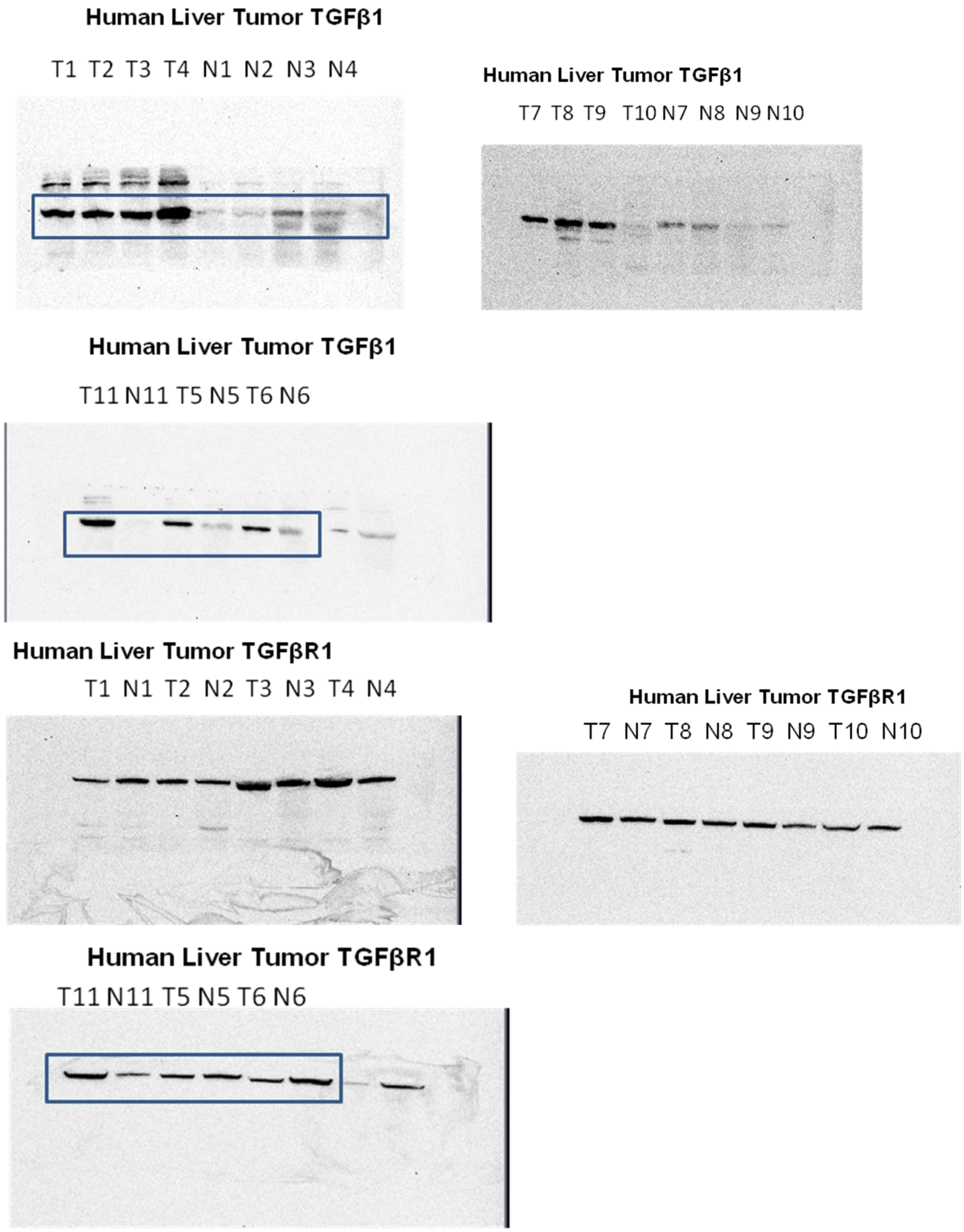

Human Liver Tumor Actin

T1 T2 T3 T4 N1 N2 N3 N4

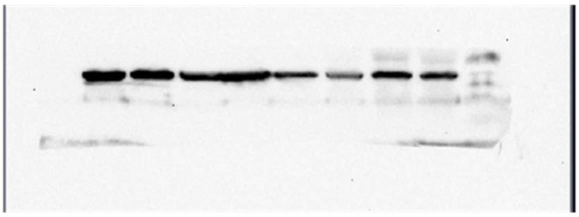

Human Liver Tumor Actin

T7 T8 T9 T10 N7 N8 N9 N10

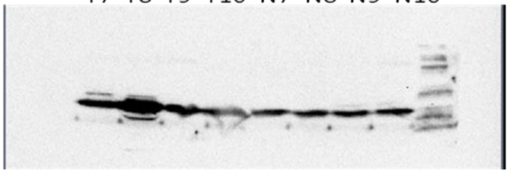

Human Liver Tumor Actin

T11 N11 T5 N5 T6 N6

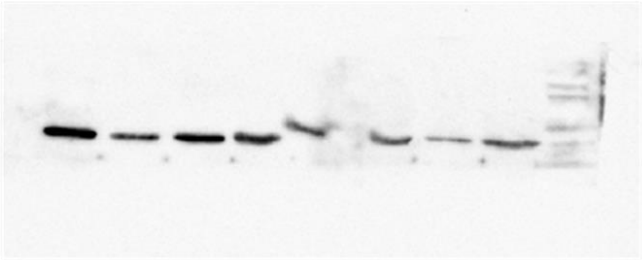

Supplementary Table S1. Conserved TGFβ1 5’UTR sequences from primates

Human	CGGGAGACCCCCAGCCCCTGCAGG
Chimp	CGGGAGACCCCCAGCCCCTGCAGG
Gorilla	CGGGAGACCCCCAGCCCCTGCAGG
Orangutan	CGGGAGACCCCCAGCCCCTGCAGG
Rhesus	CGGGAGACCCCCAGCCCCTGCAGG
Baboon	CGGGAGACCCCCAGCCCCTGCAGG
Squirrel monkey	CGCGAGACCCCCAGCCCCTGCGGG
Bushbaby	CGGGAGACCCCCAGCCCCTACAGG

Supplementary Table S2. TGFβR1 Conserved CDS sequences from 100 vertebrates

Negative	GGGGTTTGTCATGACAGCTCGTGTTGGAAA
Positive	TTAAAACACCATTGTCACAACTCAGTCAAC
Human	TTAAGAAAACATTATCGCAACTCAGTCA
Chimp	TTAAGAAAACATTATCGCAACTCAGTCA
Gorilla	TTAAGAAAACATTATCACAACTCAGTCA
Orangutan	TTAAGAAAACATTATCACAACTCAGTCA
Gibbon	TTAAGAAAACATTATCACAACTCAGTCA
Rhesus	TTAAGAAAACATTATCACAACTCAGTCA
Crab-eating macaque	TTAAGAAAACATTATCACAACTCAGTCA
Baboon	TTAAGAAAACATTATCACAACTCAGTCA
Green monkey	TTAAGAAAACATTATCACAACTCAGTCA
Marmoset	TTAAGAAAACATTATCACAACTCAGTCA
Squirrel monkey	TTAAGAAAACATTATCACAACTCAGTCA
Bushbaby	TTAAGAAAACATTGTCACAACTCAGTCA
Chinese tree shrew	TCAAGAAAACTTTGTCACAGCTCAGTCA
Squirrel	TTAAGAAGACATTGTCACAACTCAGTCA
Lesser Egyptian jerboa	TTAAAAAAACATTGTCACAGCTCAGTCA
Prairie vole	TTAAAAAAACATTGTCACAGCTCAGCCA
Chinese hamster	TTAAAAAAACATTGTCACAGCTCAGCCA
Golden hamster	TTAAAAAAACATTGTCACAGCTCAGCCA
Mouse	TTAAAAAAACATTGTCACAACTCAGCCA
Rat	TTAAAAAAACATTGTCACAGCTCAGCCA
Naked mole-rat	TTAAGAAAACATTGTCACAACTCAGTCA
Guinea pig	TTAAGAAAACATTGTCACAGCTCAGTCA
Chinchilla	TTAAGAAAACACTGTCACAACTCAGTCA
Brush-tailed rat	TTAAGAAGACACTGTCACAACTCAGTCA
Rabbit	TTAAGAAAACATTGTCACAGCTCAGTCA
Pika	TTAAGAAAACATTGTCACAGCTCAGTCA
Pig	TTAAGAAAACATTGTCACAACTCAGTCA
Alpaca	TTAAGAAAACACTGTCACAGCTCAGTCA
Bactrian camel	TTAAGAAAACATTGTCGCAGCTCAGTCA
Dolphin	TTAAGAAAACATTATCACAGCTCAGTCA
Killer whale	TTAAGAAAACATTATCACAGCTCAGTCA
Tibetan antelope	TTAAGAAAACATTGTCACAACTCAGT--
Cow	TTAAGAAAACATTGTCACAACTCAGTCA
Sheep	TTAAGAAAACATTGTCACAACTCAGTCA
Domestic goat	TTAAGAAAACATTGTCACAACTCAGTCA
Horse	TTAAGAAAACATTGTCACAGCTCAGTCA
White rhinoceros	TTAAGAAAACATTGTCACAACTCAGTCA
Cat	TTAAGAAAACATTGTCACAACTCAGTCA

Dog	TTAAGAAAACATTGTCACAACTCAGTCA
Ferret	TTAAGAAAACACTGTCACAACTCAGTCA
Panda	TTAAGAAAACATTGTCACAACTCAGTCA
Pacific walrus	TTAAGAAAACATTGTCACAACTCAGTCA
Weddell seal	TTAAGAAAACATTGTCACAACTCAGTCA
Black flying-fox	TTAAGAAAACATTGTCACAACTCAGTCA
Megabat	TTAAGAAAACATTGTCACAACTCAGTCA
David's myotis(bat)	TTAAGAAAACTTTGTCACAACTCAGTCA
Microbat	TTAAGAAAACTTTGTCACAACTCAGTCA
Big brown bat	TTAAGAAAACTTTGTCACAACTCAGTCA
Hedgehog	TTAAGAAGACATTGTCACAACTCAGTCA
Shrew	TTAAGAAGACTCTGTCACAGCTCAGCCA
Star-nosed mole	TTAAGAAAACATTGTCTCAACTCAGTCA
Elephant	TTAAGAAAACGTTATCACAACTCAGTCA
Cape elephant shrew	TTAAGAAAACGTTATCACAGCTCAGTCA
Manatee	TTAAGAAAACATTATCACAACTCAGTCA
Cape golden mole	TTAAGAAAACATTGTCACAGCTCAGCCA
Tenrec	TTAAGAAAACACTGTCTCAACTCAGTCA
Aardvark	TCAAGAAGACGTTATCACAGCTCAGTCA
Armadillo	TTAAGAAAACATTATCACAGCTCAGCCA
Opossum	TTAAGAAGACATTATCACAGCTCAGTCA
Tasmanian devil	TTAAGAAAACATTATCACAACTCAGTCA
Wallaby	TTAAGAAAACATTATCACAGCTCAGTCA
Platypus	TCAAGAAGACTCTGTCACAGCTCAGCCA
Saker falcon	TTAAGAAAACATTATCGCAACTTAGTCA
Peregrine falcon	TTAAGAAAACATTATCGCAACTTAGTCA
Collared flycatcher	TTAAGAAAACATTATCACAACTTAGTCA
White-throated sparrow	TTAAGAAAACATTATCACAACTTAGTCA
Medium ground finch	TTAAGAAAACATTATCACAACTTAGTCA
Zebra finch	TTAAGAAAACATTATCACAACTTAGTCA
Tibetan ground jay	TTAAGAAAACATTATCGCAACTTAGCCA
Budgerigar	TTAAGAAAACGTTATCACAACTTAGTCA
Parrot	TTAAGAAAACGTTATCACAACTTAGTCA
Scarlet Macaw	TTAAGAAAACGTTATCACAACTTAGTCA
Rock pigeon	TTAAGAAAACATTATCGCAACTCAGTCA
Mallard duck	TTAAGAAAACATTATCACAGCTTAGTCA
Chicken	TTAAGAAAACACTATCACAACTTAGTCA
Turkey	TTAAGAAAACACTATCACAGCTTAGTCA
American alligator	TTAAGAAAACATTATCACAGCTCAGTCA
Green seaturtle	TTAAGAAAACATTATCACAGCTCAGCCA
Painted turtle	TTAAGAAAACATTATCACAGCTCAGCCA
Soft-shell Turtle	TTAAGAAAACATTATCACAGCTCAGTCA
Spiny softshell turtle	TTAAGAAAACATTATCACAGCTCAGTCA
Lizard	TAAAGAAAACATTATCGCAGCTTAGTCA

X. tropicalis	TCAAGAAAACGTTGTCACAACTAAGCCA
Coelacanth	TGAAGAAAACACTATCACAACTGAGTCA
Tetraodon	TCAAAAAGACGCTGTCCCAGCTCAGCCA
Fugu	TCAAAAAGACGCTGTCTCAGCTCAGCCA
Tawny puffer fish	TCAAAAAGACCCTGTCGCAGCTCAGCAT
Nile tilapia	TCAAGAAAACACTGTCTCAGCTCAGCCA
Princess of Burundi	TCAAGAAAACGCTGTCTCAGCTCAGCCA
Burton's mouth breeder	TCAAGAAGACTTTGTCTCAGCTCAGCGT
Zebra Mbuna	TCAAGAAAACGCTGTCTCAGCTCAGCCA
Pundamilia nyererei	TCAAGAAAACGCTGTCTCAGCTCAGCCA
Medaka	TCAAGAAGACGCTGTCCCAGCTCAGCCA
Southern platyfish	TCAAGAAAACTGTATCTCAACT--GTCT
Stickleback	TCAAGAAGACCCTGTCTCAGCTCAGCCA
Atlantic cod	TCAAGAAGACGCTGTCCCAGCTCAGCCA
Zebrafish	TCAAGAAGTCACTGTCCCAGCTCAGTCA
Mexican tetra (cavefish)	TCAAGAAGACCCTGTCCCAGCTCAGCCA
Spotted gar	TTAAGAAGACATTGTCACAGCTCAGCCA
Lamprey	TCAAGAAGACCCTCTCGCAGCTGAGCCA

Supplementary Table S3. A list of miRNAs examined by use of SAMcell

miR-1	miR-335	miR-125b	miR-208b	miR-331-3p	miR-671-5p
miR-9	miR-340	miR-125b-3p	miR-20a	miR-331-5p	miR-708*
miR-16	miR-363	miR-126*	miR-20b	miR-335*	miR-7-1*
miR-17	miR-365	miR-127-3p	miR-214*	miR-338-3p	miR-744-3p
miR-21	miR-370	miR-127-5p	miR-216a	miR-338-5p	miR-875-5p
miR-22	miR-377	miR-129*	miR-216b	miR-33a	miR-9*
miR-24	miR-378	miR-129-3p	miR-218-2*	miR-33a*	miR-92b
miR-32	miR-379	miR-129-5p	miR-219-5p	miR-340-3p	miR-93*
miR-93	miR-381	miR-130a	miR-22-5p	miR-342-3P	miR-99a
miR-98	miR-382	miR-130b	miR-23a	miR-34a	miR-99b
miR-100	miR-410	miR-133a	miR-23b	miR-34c-5p	miR-99b*
miR-101	miR-421	miR-133b	miR-26a	miR-361-5p	let7a
miR-103	miR-425	miR-140-3p	miR-26b	miR-369-3p	let-7b
miR-107	miR-431	miR-140-5p	miR-26b*	miR-369-5P	let-7b-3p
miR-122	miR-433	miR-142-3p	miR-27a-5p	miR-374b	let-7c
miR-124	miR-448	miR-146a	miR-27b	miR-376a	let-7d
miR-126	miR-451	miR-146b-5p	miR-27b*	miR-378*	let-7d-3p
miR-128	miR-484	miR-147b	miR-296-3p	miR-378b	let-7e
miR-132	miR-494	miR-148a	miR-296-5p	miR-409-3p	let-7f
miR-137	miR-495	miR-148a*	miR-299-5p	miR-409-5p	let-7f-1-3p
miR-138	miR-496	miR-148b	miR-29a	miR-411-3p	let-7g
miR-141	miR-504	miR-151-5p	miR-29a*	miR-423-3p	let-7i
miR-143	miR-539	miR-154*	miR-29b	miR-423-5P	let7i-3p
miR-145	miR-543	miR-15a	miR-29c	miR-431-3p	miR-10a
miR-150	miR-557	miR-15b	miR-29c*	miR-449a	miR-125a-3p
miR-153	miR-568	miR-15b*	miR-301a	miR-450a	miR-134
miR-154	miR-638	miR-181a	miR-302a	miR-455-5p	miR-135b
miR-183	miR-652	miR-181a*	miR-302c	miR-485-5p	miR-139-5p
miR-184	miR-653	miR-181b	miR-302d	miR-486-5p	miR-144
miR-185	miR-663	miR-181c	miR-30a	miR-487a	miR-149
miR-186	miR-708	miR-181d	miR-30a*	miR-490-3p	miR-152
miR-187	miR-711	miR-183*	miR-30b	miR-491-5p	miR-18a
miR-190	miR-744	miR-188-3p	miR-30c	miR-499-5p	miR-190b
miR-191	miR-759	miR-188-5p	miR-30c-1*	miR-532-3p	miR-192
miR-194	miR-761	miR-193a-3p	miR-30c-2*	miR-532-5p	miR-200b
miR-195	miR-874	miR-196a	miR-30d	miR-542-3p	miR-200c
miR-204	miR-877	miR-196b	miR-30e	miR-551b	miR-203
miR-205	miR-1197	miR-199b-3p	miR-30e*	miR-574-3p	miR-25
miR-210	miR-106b	miR-199b-5p	miR-320a	miR-574-5p	miR-28-5p
miR-214	miR-106b*	miR-19a	miR-323-3p	miR-590-3p	miR-367
miR-218	miR-10a*	miR-19b	miR-323-5p	miR-590-5p	miR-7

miR-221	miR-10b	miR-200b*	miR-323b-5p	miR-615-5p	miR-760
miR-222	miR-124*	miR-200c*	miR-324-5P	miR-654-3p	
miR-223	miR-125a-5p	miR-208a	miR-330-5p	miR-671-3p	

Supplementary Table S4. miRNA hits targeting HGF or HGFR in humans or mice

miRNA	Fold change				P value			
	H-HGF	H-MET	M-HGF	M-MET	H-HGF	H-MET	M-HGF	M-MET
let-7b	0.914	0.948	1.028	0.945	0.008	0.115	0.495	0.122
let-7b-3p	1.052	0.907	0.972	0.963	0.218	0.035	0.709	0.357
let-7c	0.901	0.972	0.889	0.955	0.009	0.518	0.005	0.354
let-7d	1.046	0.919	0.905	0.973	0.219	0.013	0.027	0.344
let-7g	1.003	0.912	0.863	0.938	0.955	0.041	0.029	0.290
10a*	0.912	0.935	1.014	0.999	0.012	0.322	0.769	0.979
15b*	0.918	0.975	0.867	0.974	0.048	0.315	0.040	0.637
16	1.004	1.031	0.856	0.961	0.942	0.582	0.011	0.499
19b	0.882	1.056	1.040	0.977	0.006	0.153	0.277	0.562
20b	0.917	1.010	0.988	0.922	0.010	0.769	0.841	0.047
22-5p	0.975	0.898	0.923	0.955	0.328	0.000	0.045	0.113
24	1.014	0.921	0.932	1.009	0.722	0.032	0.127	0.830
25	0.853	0.998	0.976	0.932	0.019	0.963	0.650	0.224
26a	1.046	0.825	0.887	0.977	0.475	0.000	0.047	0.525
27b	0.920	1.005	0.966	0.984	0.036	0.924	0.396	0.669
27b*	0.796	1.062	1.046	0.964	0.003	0.125	0.233	0.215
29a*	1.113	0.962	1.026	0.922	0.005	0.314	0.574	0.026
30c	0.994	1.050	0.986	0.924	0.943	0.198	0.882	0.037
30c-1*	1.023	0.923	1.060	1.067	0.518	0.025	0.124	0.247
30e	0.899	0.975	1.032	0.934	0.011	0.630	0.392	0.416
32	0.935	0.896	0.949	1.034	0.158	0.024	0.384	0.504
33a	0.891	0.993	1.001	0.986	0.034	0.897	0.976	0.659
92b	1.005	0.889	1.031	0.992	0.917	0.001	0.397	0.867
93*	0.919	1.049	1.077	0.890	0.046	0.343	0.137	0.046
98	1.031	0.967	0.907	0.960	0.089	0.363	0.014	0.405
99a	1.065	1.041	0.793	0.975	0.063	0.137	0.000	0.543
99b*	0.849	0.970	0.999	0.898	0.002	0.652	0.980	0.022
103	0.887	0.936	1.093	1.007	0.034	0.169	0.181	0.868
106b	1.073	1.053	0.801	1.022	0.096	0.357	0.010	0.469
106b*	1.026	0.817	0.963	0.946	0.503	0.004	0.514	0.024
125a-3p	0.975	0.992	0.845	1.024	0.465	0.804	0.000	0.524
125a-5p	0.902	0.875	0.894	0.969	0.531	0.003	0.028	0.566
126*	0.920	1.027	0.839	0.992	0.018	0.631	0.001	0.813
127-5p	0.872	0.957	1.009	0.918	0.023	0.213	0.897	0.012
128	0.902	0.781	1.044	1.034	0.017	0.001	0.484	0.221
129*	1.016	0.915	1.059	1.068	0.437	0.044	0.315	0.036
129-3p	1.042	0.998	0.822	0.936	0.207	0.969	0.029	0.074
129-5p	1.029	0.885	0.948	0.935	0.576	0.002	0.289	0.060
130b	1.041	1.026	0.870	0.954	0.342	0.670	0.018	0.077
133a	0.871	0.960	0.885	0.907	0.000	0.533	0.046	0.108

137	0.919	0.934	0.937	0.922	0.007	0.100	0.091	0.046
139-5p	0.870	0.987	0.999	0.912	0.000	0.701	0.990	0.048
140-3p	1.019	1.067	0.825	0.917	0.607	0.209	0.001	0.013
141	0.905	1.029	0.932	1.001	0.046	0.693	0.296	0.980
144	0.759	1.017	0.969	0.978	0.000	0.655	0.647	0.711
145	0.881	0.962	1.098	1.010	0.017	0.363	0.099	0.777
148a	1.045	0.852	1.084	0.957	0.382	0.010	0.126	0.145
150	0.968	0.914	0.815	0.946	0.260	0.019	0.000	0.059
152	0.921	1.013	1.000	1.088	0.266	0.705	0.999	0.098
154*	0.999	0.821	0.955	0.914	0.973	0.018	0.427	0.014
181a	1.259	1.076	0.996	0.912	0.035	0.104	0.940	0.047
181a*	0.955	0.905	0.966	1.046	0.163	0.001	0.311	0.573
183*	0.899	1.014	1.024	0.975	0.013	0.770	0.544	0.519
186	0.936	1.018	0.869	0.901	0.089	0.694	0.029	0.001
188-5p	0.898	0.899	0.951	0.998	0.050	0.015	0.285	0.936
196a	1.005	0.918	0.917	0.996	0.899	0.044	0.048	0.902
196b	0.920	1.079	0.874	0.985	0.069	0.010	0.043	0.745
200b	0.869	0.983	0.938	0.982	0.013	0.617	0.428	0.699
200c*	1.073	1.041	0.823	0.964	0.031	0.289	0.032	0.279
203	1.066	0.922	1.036	1.059	0.299	0.020	0.673	0.095
208a	0.953	1.066	0.993	0.878	0.157	0.093	0.924	0.019
208b	0.890	1.127	1.032	1.018	0.016	0.328	0.566	0.622
216a	0.984	0.920	0.984	0.918	0.560	0.005	0.857	0.037
218	1.026	0.957	0.915	1.007	0.498	0.167	0.042	0.920
222	0.925	1.053	0.842	0.874	0.003	0.142	0.025	0.025
223	1.009	0.835	0.936	1.017	0.895	0.000	0.196	0.643
296-3p	0.958	1.002	0.726	0.889	0.213	0.960	0.000	0.037
301a	0.973	0.990	0.834	0.969	0.311	0.726	0.009	0.593
302a	0.946	1.042	0.903	1.055	0.104	0.765	0.044	0.436
302c	0.969	1.041	0.840	1.024	0.453	0.312	0.002	0.524
323-3p	0.993	0.936	0.884	1.032	0.803	0.101	0.012	0.285
323-5p	1.036	0.973	0.884	1.012	0.134	0.448	0.008	0.792
331-5p	0.988	0.913	0.880	1.081	0.663	0.001	0.018	0.146
335	0.954	0.965	1.026	0.894	0.284	0.138	0.677	0.004
335*	0.922	1.004	1.086	0.912	0.047	0.921	0.216	0.017
338-5p	1.034	0.906	0.964	1.014	0.422	0.001	0.429	0.790
342-3P	0.923	0.981	1.061	0.915	0.020	0.613	0.285	0.048
361-5p	0.923	0.913	0.954	0.982	0.020	0.005	0.362	0.477
363	0.834	1.005	0.966	0.985	0.019	0.870	0.445	0.591
365	0.841	0.986	0.929	0.921	0.006	0.638	0.311	0.040
369-3p	0.908	0.990	1.023	0.975	0.003	0.774	0.643	0.474
369-5P	0.955	0.921	0.983	0.921	0.401	0.008	0.753	0.032
374b	0.924	0.991	1.029	0.985	0.012	0.831	0.189	0.636
377	0.955	0.971	0.761	1.019	0.171	0.472	0.000	0.566

409-3p	0.860	1.004	1.007	0.967	0.000	0.909	0.883	0.235
410	1.066	0.882	0.944	1.048	0.068	0.031	0.251	0.106
411-3p	0.969	0.899	1.046	1.030	0.192	0.000	0.560	0.579
425	0.916	1.035	1.043	0.914	0.041	0.309	0.270	0.033
431	0.917	1.023	0.966	1.033	0.049	0.616	0.624	0.442
431-3p	0.908	0.952	1.001	0.842	0.005	0.291	0.991	0.016
448	0.932	0.940	0.818	1.007	0.030	0.097	0.003	0.834
450a	0.954	0.962	0.982	1.033	0.147	0.231	0.774	0.416
455-5p	0.884	0.942	0.854	0.936	0.008	0.249	0.005	0.086
490-3p	1.032	0.959	0.912	0.934	0.064	0.193	0.009	0.077
495	1.048	0.989	0.902	0.940	0.155	0.831	0.037	0.131
496	0.981	0.904	0.922	0.947	0.550	0.004	0.003	0.358
504	0.850	0.965	1.055	0.973	0.015	0.387	0.248	0.549
539	0.920	1.052	1.027	0.983	0.015	0.123	0.657	0.658
542-3p	0.960	0.984	1.056	0.900	0.322	0.581	0.207	0.026
574-5p	0.994	1.011	1.063	0.906	0.899	0.692	0.138	0.019
590-5p	1.019	0.855	0.986	1.024	0.620	0.002	0.822	0.544
652	1.037	0.989	0.925	0.952	0.058	0.716	0.036	0.266
653	0.896	0.995	0.969	0.864	0.005	0.908	0.578	0.018
708	0.838	1.014	0.989	0.950	0.004	0.705	0.895	0.091
708*	1.008	0.909	0.777	0.955	0.724	0.005	0.000	0.281
744-3p	0.898	0.857	0.976	1.084	0.009	0.000	0.683	0.315
761	0.944	0.913	0.963	0.957	0.566	0.031	0.487	0.310
1197	0.914	1.128	0.814	0.958	0.037	0.015	0.003	0.495

Note: Hits with cutoff of 0.92 and p-value <0.05 were highlighted in red.

Supplementary Table S5. miRNA hits targeting FGF or FGFR in humans or mice

miRNAs	Fold change				P value			
	H-FGF1	H-FGFR1	M-FGF1	M-FGFR1	H-FGF1	H-FGFR1	M-FGF1	M-FGFR1
let-7b	0.941	1.055	0.843	0.980	0.323	0.402	0.028	0.533
let-7b-3p	0.977	1.015	1.082	0.849	0.681	0.642	0.273	0.018
let-7f-1-3p	0.958	1.030	0.794	0.948	0.409	0.555	0.002	0.373
let-7i	0.889	0.915	1.051	0.944	0.082	0.023	0.159	0.042
let-7i-3p	0.860	0.989	0.855	1.053	0.028	0.775	0.025	0.333
7	0.942	0.892	1.046	1.023	0.081	0.041	0.195	0.547
10a	0.997	0.952	0.866	0.992	0.931	0.080	0.047	0.892
19a	1.057	0.908	0.973	0.984	0.385	0.004	0.615	0.732
22-5p	0.918	0.914	0.917	0.946	0.153	0.014	0.015	0.301
25	0.887	0.959	1.096	1.062	0.022	0.103	0.083	0.133
26a	0.933	1.109	0.937	0.882	0.274	0.092	0.077	0.003
26b	0.892	1.004	0.912	1.079	0.007	0.911	0.019	0.119
27a-5p	0.922	1.013	0.985	1.054	0.037	0.719	0.700	0.046
29a	1.005	0.919	0.916	0.906	0.900	0.106	0.030	0.046
29a*	0.917	1.006	0.915	1.066	0.002	0.907	0.038	0.145
29b	1.020	0.933	0.979	0.884	0.564	0.122	0.720	0.032
29c	1.008	0.891	1.095	0.941	0.845	0.039	0.159	0.004
30c-2*	0.936	0.902	1.023	0.969	0.020	0.032	0.427	0.368
32	0.914	0.995	1.002	1.005	0.031	0.955	0.957	0.833
34c-5p	1.129	1.006	1.003	0.878	0.017	0.848	0.954	0.002
92b	1.045	0.840	0.990	1.045	0.212	0.000	0.771	0.264
93*	0.910	1.118	0.944	0.979	0.001	0.090	0.070	0.503
103	0.914	0.919	1.108	0.862	0.008	0.185	0.346	0.002
107	1.001	0.893	1.017	0.904	0.972	0.008	0.618	0.003
125a-3p	1.084	0.919	0.971	0.771	0.012	0.017	0.357	0.004
125b	0.972	0.895	0.903	0.989	0.565	0.045	0.021	0.730
125b-1-3p	0.857	1.073	1.044	0.933	0.001	0.164	0.243	0.208
128	0.918	0.973	0.979	0.843	0.035	0.441	0.715	0.005
129-5p	0.929	1.159	0.922	0.906	0.298	0.029	0.062	0.013
129*	0.894	0.995	0.931	0.918	0.012	0.893	0.114	0.049
133a	1.060	1.053	0.861	0.973	0.301	0.172	0.043	0.522
135b	0.982	0.842	0.917	0.908	0.545	0.004	0.049	0.300
137	0.913	0.936	0.992	0.909	0.027	0.250	0.861	0.018
139-5p	0.825	1.026	0.911	0.987	0.001	0.487	0.025	0.640
140-5p	0.858	0.948	1.045	1.072	0.001	0.077	0.603	0.610
144	0.864	0.980	1.011	1.014	0.002	0.523	0.799	0.641
145	0.870	0.905	0.964	0.964	0.000	0.005	0.494	0.498
146b-5p	1.079	0.994	0.973	0.912	0.103	0.873	0.588	0.017
153	0.903	1.011	1.069	1.042	0.025	0.837	0.040	0.205
181a	1.055	0.913	0.951	1.100	0.229	0.018	0.269	0.171

181a*	0.899	0.984	0.950	0.953	0.016	0.785	0.131	0.254
181c	1.039	0.904	1.056	1.021	0.511	0.003	0.319	0.596
190b	0.896	0.976	1.017	0.984	0.006	0.636	0.551	0.793
192	0.903	0.992	1.088	0.945	0.011	0.850	0.080	0.401
193a-3p	0.902	0.959	0.957	0.904	0.042	0.230	0.462	0.048
199b-5p	0.931	0.901	0.947	0.938	0.045	0.015	0.294	0.119
200c	0.881	1.027	1.112	1.068	0.003	0.463	0.001	0.414
203	0.947	0.969	0.828	1.016	0.175	0.295	0.018	0.718
204	1.063	0.848	0.942	0.951	0.143	0.047	0.103	0.409
205	1.037	0.953	1.024	0.863	0.379	0.301	0.516	0.024
208b	0.882	0.920	0.924	0.875	0.010	0.002	0.207	0.003
214*	1.106	0.964	0.992	0.920	0.037	0.235	0.855	0.019
216a	0.906	0.913	0.949	0.975	0.121	0.014	0.205	0.562
218	0.914	1.100	0.917	1.048	0.013	0.122	0.032	0.179
219-5p	0.912	1.024	0.944	0.895	0.012	0.623	0.136	0.041
223	0.859	0.962	0.965	0.996	0.030	0.555	0.298	0.866
296-5p	0.916	0.969	0.966	0.748	0.034	0.301	0.310	0.004
302a	0.917	0.963	0.872	0.902	0.032	0.197	0.014	0.014
302c	0.994	0.893	1.053	0.978	0.875	0.047	0.216	0.315
335*	1.067	0.920	1.000	0.994	0.015	0.026	0.990	0.843
338-5p	0.953	0.906	0.954	1.061	0.134	0.018	0.136	0.152
361-5p	0.924	0.893	0.896	0.848	0.092	0.113	0.008	0.021
377	1.038	0.937	1.026	0.875	0.373	0.295	0.495	0.023
381	1.016	0.957	0.969	0.871	0.695	0.200	0.478	0.000
409-3p	1.014	0.909	0.916	1.052	0.674	0.044	0.041	0.089
409-5p	0.943	0.876	0.964	0.935	0.171	0.014	0.358	0.273
411-3p	0.885	0.988	1.045	0.939	0.036	0.770	0.616	0.333
448	0.959	0.883	1.008	1.071	0.404	0.031	0.835	0.194
450a	1.028	1.033	1.057	0.877	0.769	0.427	0.346	0.011
494	0.915	0.995	1.021	0.877	0.010	0.800	0.768	0.002
496	0.887	0.890	0.997	0.954	0.003	0.012	0.943	0.362
504	0.920	0.980	1.068	1.018	0.045	0.599	0.054	0.693
532-3p	0.986	0.856	0.955	0.914	0.673	0.003	0.159	0.123
551b	1.037	0.915	0.985	1.029	0.428	0.026	0.662	0.651
557	0.917	0.977	0.992	0.894	0.037	0.635	0.811	0.003
615-5p	0.982	0.976	0.883	0.916	0.746	0.293	0.049	0.025
652	1.089	0.887	0.983	1.127	0.005	0.009	0.709	0.010
663	0.905	0.945	0.940	1.011	0.036	0.213	0.033	0.778
671-3p	0.975	0.914	0.980	1.044	0.502	0.044	0.614	0.468
671-5p	0.894	1.053	0.906	0.855	0.011	0.424	0.174	0.006
708	1.003	0.890	1.068	0.909	0.927	0.030	0.056	0.031
708*	1.031	0.869	0.966	1.117	0.424	0.004	0.416	0.029
744	0.906	0.943	0.941	0.911	0.000	0.274	0.189	0.012
874	0.950	1.029	0.883	1.001	0.192	0.532	0.043	0.985

Note: Hits with cutoff of 0.92 and p-value <0.05 were highlighted in red.

Supplementary Table S6. miRNA hits targeting TGFβ1 or TGFβR1 in humans or mice

miRNAs	Fold change				
	H-TGFβ1	H-TGFβR1-A	H-TGFβR1-B	M-TGFβ1	M-TGFβR1
let7a	0.846	0.990	0.982	0.958	0.980
let-7b-3p	1.014	0.980	0.958	0.789	0.967
let-7c	1.024	1.026	0.979	0.929	0.910
let-7d	1.005	0.981	0.970	0.867	1.014
let-7e	1.056	0.942	0.920	0.909	1.062
let-7f	0.984	1.018	0.952	0.860	1.048
let-7g	1.143	0.984	0.910	0.870	0.921
let-7i	0.930	0.903	0.949	0.980	0.919
7	0.817	1.013	0.988	0.933	0.919
10a	0.865	0.959	0.983	0.979	1.051
15b	0.994	0.919	0.970	0.969	0.947
20b	0.932	0.964	0.941	0.895	0.928
24	1.092	1.033	1.027	0.913	0.982
26a	0.929	0.996	1.021	0.886	0.967
26b	0.963	1.002	1.044	0.855	1.012
26b*	0.866	0.972	0.980	1.023	0.983
27b	0.985	0.965	1.006	0.942	0.919
29c*	0.933	1.011	0.978	0.861	0.976
30a	0.869	0.964	0.991	1.091	1.029
30c	0.898	0.993	1.024	0.985	1.013
30c-2*	1.081	0.960	1.009	0.914	0.931
30e	1.002	0.963	1.085	0.880	1.094
92b	1.014	0.964	0.892	0.847	1.002
93	0.870	0.969	0.972	1.213	1.022
99a	0.939	0.944	0.969	0.878	0.961
103	1.011	1.056	0.988	0.816	1.006
107	1.023	1.009	0.905	0.808	1.059
124*	0.854	0.993	1.005	0.945	0.991
125a-5p	1.014	0.980	1.077	0.860	0.955
125b	1.008	0.973	1.000	0.988	0.905
126	0.902	0.910	0.963	0.977	0.993
127-5p	1.029	1.077	1.028	1.062	0.900
129*	0.908	0.961	1.033	1.002	0.944
129-3p	0.951	1.008	0.939	0.744	0.974
140-5p	0.920	0.984	0.955	0.934	0.927
145	0.860	1.014	0.998	0.823	1.065
151-5p	1.025	0.986	0.913	1.068	1.066
154*	0.957	1.017	1.058	0.857	0.996
181a*	0.772	0.909	0.893	0.906	0.917
181b	1.064	0.984	0.900	0.938	1.033

185	1.148	0.971	0.876	1.019	0.960
191	0.962	1.004	0.997	0.881	0.987
193a-3p	1.021	0.873	1.092	0.828	1.029
195	1.050	0.985	1.032	0.909	1.004
196a	0.886	0.944	1.010	1.182	1.026
196b	0.862	1.018	0.996	1.035	1.004
200b	0.866	1.024	0.959	0.976	1.036
208b	0.996	0.944	1.017	0.909	1.039
216a	1.007	0.876	1.060	0.892	1.049
218	0.979	0.989	1.035	0.829	0.963
221	1.076	0.956	0.878	1.008	0.983
296-3p	1.100	0.989	1.038	0.887	0.957
301a	1.063	0.905	0.930	1.024	0.984
302a	1.017	1.022	0.948	0.994	0.837
323-3p	0.925	1.046	1.034	0.831	0.986
324-5P	1.036	0.893	0.989	0.988	1.019
331-3p	0.997	0.918	0.985	0.847	1.085
335	1.005	0.905	1.003	0.945	0.973
335*	0.976	0.883	0.946	0.955	1.038
338-5p	0.936	1.075	0.916	0.892	1.007
342-3P	1.023	0.890	0.922	0.966	0.943
365	0.880	0.956	0.934	0.982	1.058
369-3p	0.877	0.985	1.004	0.948	1.046
374b	1.002	0.966	1.087	0.841	0.953
377	0.955	0.935	1.004	0.850	0.979
378b	1.035	0.914	1.082	1.071	1.052
381	0.873	1.001	0.927	0.931	0.987
382	0.950	1.005	1.002	0.895	1.029
410	1.044	1.002	1.008	0.880	0.910
411-3p	0.972	0.891	1.059	0.790	0.999
421	0.859	0.990	0.985	0.916	0.880
448	1.034	0.922	0.922	0.756	1.125
487a	1.067	0.905	1.031	1.084	0.986
490-3p	0.972	0.999	1.086	0.822	0.994
495	0.933	0.951	0.969	0.831	1.000
496	0.900	0.970	1.018	0.808	0.926
499-5p	0.911	1.016	1.004	1.008	0.997
532-5p	0.980	0.962	1.061	0.848	0.986
543	1.090	1.008	1.015	0.909	0.910
568	0.834	1.003	0.996	1.085	0.957
574-3p	0.873	0.970	1.035	0.971	1.061
663	0.904	0.948	0.969	0.888	0.972
708	1.115	0.962	0.979	1.222	0.920

708	1.069	1.021	1.069	1.047	0.906
708*	0.843	0.953	1.008	0.771	0.975
711	1.060	1.034	0.980	0.955	0.878
761	0.939	0.919	1.026	0.868	1.086
875-5p	0.830	0.997	0.992	1.064	0.956

miRNAs	P value				
	H-TGFβ1	H-TGFβR1-A	H-TGFβR1-B	M-TGFβ1	M-TGFβR1
let7a	0.000	0.622	0.594	0.352	0.815
let-7b-3p	0.728	0.323	0.292	0.001	0.411
let-7c	0.645	0.408	0.456	0.095	0.002
let-7d	0.915	0.336	0.430	0.000	0.601
let-7e	0.036	0.005	0.007	0.017	0.192
let-7f	0.639	0.445	0.241	0.028	0.375
let-7g	0.010	0.534	0.008	0.030	0.009
let-7i	0.153	0.004	0.150	0.404	0.041
7	0.001	0.502	0.748	0.123	0.010
10a	0.003	0.106	0.597	0.667	0.088
15b	0.915	0.001	0.315	0.343	0.171
20b	0.058	0.232	0.027	0.021	0.029
24	0.140	0.065	0.444	0.011	0.519
26a	0.057	0.775	0.535	0.004	0.094
26b	0.378	0.914	0.061	0.000	0.697
26b*	0.049	0.188	0.475	0.696	0.634
27b	0.705	0.057	0.863	0.283	0.007
29c*	0.099	0.542	0.396	0.002	0.218
30a	0.047	0.119	0.605	0.063	0.190
30c	0.048	0.809	0.463	0.774	0.556
30c-2*	0.205	0.214	0.631	0.031	0.001
30e	0.959	0.107	0.032	0.012	0.026
92b	0.772	0.231	0.001	0.049	0.944
93	0.019	0.048	0.251	0.000	0.271
99a	0.141	0.022	0.274	0.037	0.028
103	0.849	0.016	0.724	0.005	0.751
107	0.565	0.751	0.002	0.009	0.079
124*	0.006	0.766	0.846	0.111	0.840
125a-5p	0.795	0.435	0.040	0.043	0.070
125b	0.844	0.161	0.998	0.800	0.016
126	0.126	0.000	0.139	0.704	0.001
127-5p	0.594	0.012	0.107	0.237	0.000
129*	0.007	0.158	0.151	0.959	0.137
129-3p	0.033	0.685	0.017	0.000	0.268
140-5p	0.045	0.596	0.108	0.355	0.030

145	0.000	0.395	0.941	0.000	0.001
151-5p	0.652	0.400	0.014	0.479	0.006
154*	0.303	0.619	0.096	0.026	0.851
181a*	0.000	0.013	0.001	0.040	0.015
181b	0.033	0.427	0.006	0.367	0.497
185	0.039	0.141	0.002	0.720	0.057
191	0.295	0.891	0.933	0.014	0.611
193a-3p	0.627	0.000	0.003	0.017	0.460
195	0.251	0.349	0.280	0.034	0.867
196a	0.000	0.028	0.676	0.001	0.421
196b	0.015	0.207	0.848	0.671	0.782
200b	0.001	0.274	0.162	0.608	0.075
208b	0.920	0.017	0.587	0.029	0.266
216a	0.845	0.001	0.034	0.036	0.079
218	0.723	0.594	0.066	0.000	0.123
221	0.120	0.085	0.009	0.837	0.427
296-3p	0.056	0.498	0.341	0.039	0.010
301a	0.061	0.000	0.014	0.597	0.384
302a	0.669	0.416	0.145	0.914	0.000
323-3p	0.040	0.006	0.341	0.005	0.348
324-5P	0.459	0.005	0.750	0.786	0.542
331-3p	0.929	0.010	0.616	0.010	0.015
335	0.933	0.009	0.925	0.297	0.420
335*	0.706	0.001	0.207	0.409	0.262
338-5p	0.110	0.179	0.047	0.022	0.725
342-3P	0.558	0.002	0.020	0.407	0.014
365	0.001	0.044	0.132	0.776	0.222
369-3p	0.046	0.509	0.863	0.309	0.052
374b	0.986	0.125	0.020	0.011	0.040
377	0.195	0.027	0.903	0.001	0.516
378b	0.227	0.000	0.041	0.058	0.004
381	0.002	0.970	0.052	0.041	0.522
382	0.179	0.842	0.936	0.031	0.544
410	0.330	0.898	0.793	0.014	0.041
411-3p	0.464	0.000	0.059	0.002	0.976
421	0.002	0.674	0.682	0.053	0.012
448	0.393	0.005	0.020	0.000	0.009
487a	0.148	0.000	0.379	0.059	0.520
490-3p	0.502	0.982	0.012	0.002	0.761
495	0.082	0.050	0.271	0.003	0.984
496	0.039	0.312	0.463	0.000	0.000
499-5p	0.037	0.319	0.920	0.785	0.873
532-5p	0.706	0.106	0.053	0.009	0.333
543	0.069	0.472	0.616	0.032	0.008

568	0.012	0.869	0.883	0.102	0.273
574-3p	0.004	0.229	0.169	0.576	0.010
663	0.041	0.083	0.201	0.026	0.197
708	0.034	0.116	0.706	0.005	0.025
708	0.117	0.133	0.011	0.121	0.049
708*	0.001	0.071	0.798	0.000	0.126
711	0.125	0.320	0.358	0.532	0.000
761	0.264	0.003	0.448	0.017	0.039
875-5p	0.001	0.841	0.733	0.532	0.081

Note: Hits with cutoff of 0.92 and p-value <0.05 were highlighted in red.

Supplementary Table S7. Primer list of genes to be cloned

Description 1	5'-3' Sequence
Human *TGFβ1* 5'UTR	GGGCCATCTCCCTCCCAC
	ACTGCCGAGAGCGCGAAC
Human *TGFβR1* 3'UTR 1-2600	TTCTACAGCTTTGCCTGAACTC
	GAAGATGTTTTAGCCACGTTGA
Human TGFβR1 3'UTR 2500-4600	GGATGGATCATGATTACTGTCG
	GTTCTCTTGTAAGCCAAGTTTTCAC
Human *TGFβ1* coding region*	CGCAGTCACCGTCCTGACACG
	CGGCGATCCTCTGGAGATTG
Monkey *TGFβ1* 5'UTR	GGCCCCAGAGTCTGAGACGA
	TGCCGAGAGGGCGAACAG
Monkey *TGFβR1* 3'UTR	CCCAGGAAACAGCCGTGT
	CAAGATTGCATAACGGGATTGA
Mouse *TGFβ1* 5'UTR	GGATCCTCCAGACAGCCAGG
	CACTTCGGAGAGCGGGAAC
Mouse *TGFβR1* 3'UTR	CTGAAACACCGTGGGAACTCTGCTCTCTTC
	CGCGATGATGTCATTTGGGGTACAGATATAAAC
Rat *TGFβR1* 3'UTR	TACTTCTTCCCAGACAGTTACAGA
	CTGCACTCATTCCCAGACTAC
Human *FGF1* 3'UTR	CTCCCCAGACTCCACCGTC
	ATGCCCTGGGAGAAGATACAG
Mouse *FGF1* 3'UTR	CTTGACCATTGGCTGCGCTAA
	CCCGGATGGTGACTAGGTTGA
Human *FGFR1* 3'UTR	CTCCCCAGACTCCACCGTC
	AAGACACTCCTGTGCGGTCTC
Mouse *FGFR1* 3'UTR	TCTCCCATTCCGTCGTCAC
	TGCATTTAAAGAAGCAGCCCT
Human *HGF* 3'UTR	CCCCTATGTGTAGAACTGTAAC
	TAGGGAACAGAATATGATATG
Mouse *HGF* 3'UTR	CCAGTGTGTTTGAAGCATCCA
	CTGGAAAGCAGCATAGAGTGA
Human *HGFR* 3'UTR	GCCAAAATTGCACTATTATAGG
	TTCTTTCTATTGATGCGTTCAT
Mouse *HGFR* 3'UTR	CCCAAACCAGACTATCCC
	TGCCCTAGGAAGAGTCATAA

Note:

1. Other 5'UTRs or 3'UTRs from TGFβ1 or TGFβR1 not described above were synthesized according to the sequences published in GenBank.

*: Cloned from TGFβ1 plasmid purchased from Sino Biological Inc.
